# Effects of Etching and Delamination on Biocompatibility of Ti-Based MXenes

**DOI:** 10.1021/acsami.5c08807

**Published:** 2025-08-18

**Authors:** Kateryna Diedkova, Iryna Roslyk, Nikola Kanas, Lita Grine, Volodymyr Deineka, Agata Blacha-Grzechnik, Martins Boroduskis, Igor Iatsunskyi, Błażej Anastaziak, Anastasia Konieva, Pavlo Shubin, Wojciech Simka, Marks Truhins, Oksana Sulaieva, Ilya Yanko, Veronika Zahorodna, Goran Stojanovic, Oleksiy Gogotsi, Yury Gogotsi, Maksym Pogorielov

**Affiliations:** † Biomedical Research Centre, 187506 Sumy State University, Sumy 40007, Ukraine; ‡ 61769 University of Latvia, Riga LV-1004, Latvia; § A. J. Drexel Nanomaterials Institute, and Department of Materials Science and Engineering, 6527 Drexel University, Philadelphia, Pennsylvania 19104, United States; ∥ 84981 University of Novi Sad, Zorana Dindica 1, Novi Sad 21000, Serbia; ⊥ Faculty of Chemistry, 49569 Silesian University of Technology, Strzody 9, Gliwice 44-100, Poland; # Centre for Organic and Nanohybrid Electronics, Silesian University of Technology, Konarskiego 22B, Gliwice 44-100, Poland; ∇ NanoBioMedical Centre, 49562 Adam Mickiewicz University, 3, Wszechnicy Piastowskiej Str., Poznan 61-614, Poland; ○ Department of Anatomy, 39081 University Hospital Essen, Hufelandstraße 55, Essen 45147, Germany; ◆ Medical Laboratory CSD, 45 Vasylkivska St, Kyiv 02000, Ukraine; ¶ Materials Research Centre, 3 Krzhizhanovskogo Str., Kyiv 03142, Ukraine

**Keywords:** MXenes, surface terminations, etching, intercalation, biocompatibility, in vivo tolerance, toxicity mechanism

## Abstract

MXenes, a class of two-dimensional transition metal carbides and nitrides, have emerged as promising candidates for biomedical applications due to their electrical conductivity, photothermal response, and rich surface chemistry. However, their biocompatibility is highly sensitive to synthesis conditions, particularly etching and delamination strategies. In this study, we systematically investigated the influence of different synthesis routes—using acidic (concentrated or diluted HF/HCl) etching and Li^+^ versus Na^+^ intercalation—on the surface chemistry, structural integrity, and biological behavior of Ti_3_C_2_T_*x*_ and its carbonitride analog Ti_3_C_1.5_N_0.5_T_*x*_. Detailed physicochemical characterization revealed that water-assisted etching and Na^+^ intercalation enhanced hydroxylation and reduced fluorine terminations. Biological assays using human keratinocytes (HaCaT) demonstrated that Ti_3_C_1.5_N_0.5_T_*x*_ exhibited superior biocompatibility compared to Ti_3_C_2_T_*x*_, with lower cytotoxicity, diminished ROS generation, minimal inflammatory signaling (IL-6 and IL-8 interleukins), and preserved wound healing capacity. Among Ti_3_C_2_T_*x*_ variants, the combination of diluted etchant and Na^+^ intercalation significantly improved biological tolerance, minimizing apoptosis and oxidative stress. These findings underscore the critical role of surface chemistry in MXene-cell interactions and offer a practical guide to engineering safer MXenes for biomedical use.

## Graphical abstract

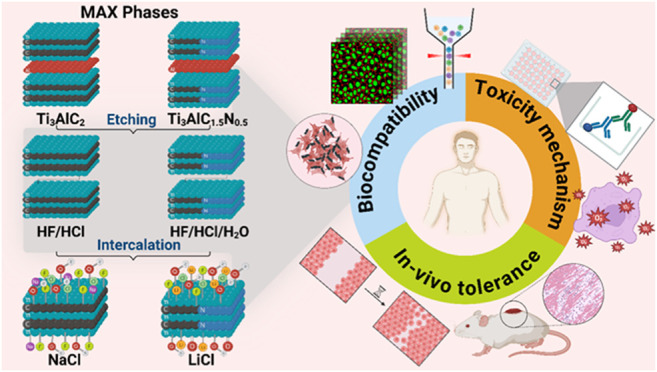

## Introduction

MXenes—a family of two-dimensional transition metal carbides, nitrides, and carbonitrides–have emerged as uniquely versatile materials with chemically tunable surfaces.^1,2^ Unlike graphene (with largely inert basal planes) or poorly conductive oxide nanosheets, MXenes combine high electrical conductivity with hydrophilicity and rich surface chemistry. Their general formula M_*n*+1_X_*n*_T_*x*_ highlights this tunability: an early transition metal layer (M) bonded to carbon and/or nitrogen (X) is terminated by surface functional groups (T_*x*_ is −OH, =O, halogen, chalcogen, etc.) arising from synthesis or post-treatment.^3^ These surface terminations— and thus the material’s composition and interfacial properties—are not intrinsic constants; they are shaped by how we etch and delaminate the MXene from its MAX phase precursor. In essence, how a MXene is born determines how it interacts with the world, especially with biological systems.^4^

Synthesis routes to MXenes can alter their surface chemistry and morphology. The traditional preparation of Ti_3_C_2_T_*x*_ involves acidic etchants like HF or in situ HF (from LiF + HCl), which efficiently remove the Al layer from Ti_3_AlC_2_ but leave behind a partially fluorinated Ti_3_C_2_ surface decorated with −F alongside −OH/–O terminations.^5,6^ The fluorine coverage and density of point defects depend on HF concentration in the etchant and the composition of the etching solution, in general.^7,8^ By contrast, emerging fluoride-free aqueous etching methods yield different surface functionalizations. For example, a recent study demonstrated that using a NaOH hydrothermal etch (a strongly alkaline, water-based route) produces Ti_3_C_2_T_*x*_ with exclusively −O/–OH terminations (essentially halogen-free).^9^ These etchant-driven differences in terminations profoundly influence MXene properties: the NaOH-etched (halogen-free) Ti_3_C_2_ showed a red-shifted optical absorption peak and far more hydrophilic surface than its HF-etched counterpart, consistent with a higher −O/–OH content. Moreover, alternative etchants can introduce other terminal groups; for instance, using elemental halogens or molten salts in etching can yield chlorine- or iodine-terminated MXenes.^10^ Such surface functionalization routes are not mere chemical curiosities—they dictate how MXene flakes disperse in water, how stable they are against oxidation, and how they interface with biological molecules.

Beyond etching, the delamination and intercalation strategy used to exfoliate multilayered MXenes into single-layer nanosheets is another key factor governing their structure and biointeractions. Common delamination techniques involve inserting cations or molecules between MXene layers to weaken interlayer bonds. Lithium ions (Li^+^) introduced via Li-salts (e.g., LiCl or LiF in HCl) are frequently used to achieve high-quality Ti_3_C_2_T_*x*_ colloidal suspensions.^11^ However, recent insights suggest this choice may have biological repercussions: machine-learning analyses of MXene cytotoxicity identified residual Li^+^ on MXene surfaces as a potential driver of cell toxicity.^3^ In other words, Li^+^ intercalation—if not followed by thorough purification—could leave behind cytotoxic Li residues on Ti_3_C_2_T_*x*_ flakes. This has prompted interest in using cations like Na^+^ as alternative intercalants. Indeed, MXenes can be delaminated with Na^+^ or K^+^ under modified conditions,^12^ and different intercalant ions are known to influence the hydration behavior and stability of the resulting MXene films. For example, Na-intercalated Ti_3_C_2_T_*x*_ may retain a thicker water layer between sheets, potentially improving colloidal stability and reducing restacking, which in turn could affect how cells encounter these nanosheets. NaCl is biocompatible, environmentally friendly, and less expensive than the commonly used LiCl. Despite these observations, systematic comparisons of Li^+^ vs Na^+^ (and their respective intercalation byproducts) on MXene biocompatibility are lacking.

Across the literature, Ti_3_C_2_T_*x*_ MXenes generally demonstrate high cell viability at low-to-moderate concentrations, with cytotoxicity becoming significant only at higher doses or after long exposure. For example, multiple studies have shown >70–80% viability in various human cell lines at 50–100 μg/mL of Ti_3_C_2_T_*x*_. At higher concentrations (e.g., > 200 μg/mL), some reduction in metabolic activity is observed, often dose-dependent. Oxidative stress is a commonly observed cellular response to MXene exposure. Elevated reactive oxygen species (ROS) levels have been measured in cells after MXene treatment, especially at sublethal doses. This ROS generation can trigger mitochondrial dysfunction and apoptotic pathways. Initial studies of Ti_3_C_2_T_*x*_ in vitro raised red flags by showing that MXene flakes (synthesized via conventional methods) could induce significant oxidative stress in cells. For instance, Jastrzębska et al. observed elevated reactive oxygen species (ROS) in cells exposed to Ti_3_C_2_T_*x*_, correlating with cytotoxic effects (interestingly, more pronounced in cancer cells than in healthy ones). Such oxidative stress is often a surface-driven phenomenon: as MXene surfaces oxidize or leach ions, they can generate ROS or disrupt cellular redox balance. Subtle changes in surface chemistry can tip the balance – the presence of surface Ti­(III) or slightly oxidized Ti_3_C_2_ domains was found to noticeably increase ROS generation compared to pristine Ti_3_C_2_T_*x*_. Likewise, as mentioned above, residual etchant or intercalant species (e.g., Li^+^) or certain terminations (e.g., −F) may provoke inflammation or toxicity. On the other hand, there is evidence that strategically engineered MXenes can be quite biocompatible. Yoon et al. recently showed that a Ti_3_C_2_T_*x*_ produced via halogen-free (NaOH) etching caused no significant cytotoxicity even at 2 mg/mL, whereas a standard HF-etched Ti_3_C_2_T_*x*_ control led to ∼ 50% cell viability loss at the same high dose. This was attributed to the fluorinated MXene releasing harmful species upon hydrolysis. This underscores how MXene synthesis methods can make a difference between a biofriendly nanomaterial and a cytotoxic one. Ti_3_C_2_T_*x*_ MXene’s biocompatibility is tunable by synthesis parameters—using etching protocols that reduce residual fluoride (or eliminate halogens entirely) tends to yield MXenes that are more biocompatible and cause less oxidative stress in cells. Delamination methods that produce well-dispersed, smaller flakes (without excessive damage to the MXene or introducing toxic ions) can minimize acute cytotoxicity and inflammation. Flake size and thickness have nuanced effects: intermediate-sized, monolayer MXenes are generally benign to cells, whereas extremely small or very large flakes can introduce unique stresses (chemical and physical, respectively). Crucially, most studies concur that MXenes exhibit low in vitro toxicity at doses under ∼ 50 μg/mL, and even at higher exposures, cell viability often remains above 70% for many cell types. End points like cell metabolism, membrane integrity, and apoptosis show dose-dependent but manageable changes, with some indication of ROS generation as the primary mechanism driving any MXene-induced cytotoxicity. Inflammatory and immune activation markers (IL-6 and IL-8 interleukins) are largely unchanged by pristine MXenes, aside from slight increases linked to larger flake stimuli. Lastly, initial investigations into genotoxicity find no inherent DNA damage caused by MXenes, reinforcing the view that with proper synthesis and handling, Ti_3_C_2_T_*x*_ MXene can be rendered biocompatible for biomedical use.^13^ The current literature review shows that controlling surface terminations and particle size during MXene synthesis is key to optimizing their safety profile for biological applications. But, the field still lacks unified guidelines on which MXene formulations are optimal for biological use, partly because the synthesis–bioperformance relationship has not been systematically explored.

As MXenes move toward applications in biosensing, drug delivery, tissue engineering, photothermal therapy, and other biomedical arenas,^14,15^ there is a pressing need to connect the chemical “dots” of MXene processing with the biological “outcomes” in cells and tissues. Early reviews have flagged the “lack of knowledge” in this area as a major obstacle to safe MXene deployment.^16^ Researchers have noted variations in toxicity with MXene stoichiometry (e.g., Ti_3_C_2_T_*x*_ vs Ti_2_CT_*x*_) and layer thickness,^17^ implying that composition and exfoliation extent matter, but comprehensive data connecting each synthesis step (etchant choice, intercalant type, etc.) to biocompatibility is still missing. Ti_3_C_2_T_*x*_ remains the most-studied MXene, whereas Ti_3_C_1.5_N_0.5_T_*x*_ (a mixed carbide-nitride) represents a newer, less-explored composition that nominally has the same surface chemistry, but different X-sublattice composition and electronic structure,^18^ which alter its properties.^19,20^ Comparing these MXenes could reveal whether nitrogen incorporation modulates surface functionalization or biological response—an intriguing question given that nitrides might interact differently with water or cell environments. To date, however, systematic comparisons of carbide vs carbonitride MXenes under varying synthesis conditions have not been reported, leaving a niche of scientific uncertainty.

In light of these challenges and knowledge gaps, the present study systematically explores how MXene synthesis influences biological responses. We focus on two representative MXenes—Ti_3_C_2_T_*x*_ ^11^ and Ti_3_C_1.5_N_0.5_T_*x*_ ^18^—and subjected each to a range of etching and delamination protocols (varying the acid ratio and concentration, and Li^+^ vs Na^+^ intercalation routes). By characterizing the resulting differences in surface termination composition, layer morphology, and colloidal stability, and correlating these with a battery of biological assays (assessing cell viability, cytotoxicity, and oxidative stress indicators), we aim to illuminate the synthesis–biocompatibility link in a rigorous, comparative manner. This work addresses the gap in MXene research regarding processing-dependent bioperformance. The insights gleaned here will not only reconcile some of the inconsistent reports in the literature but also provide practical guidance for designing MXenes with safer biological profiles. In bridging the chemistry of MXene synthesis with the intricacies of biological interaction, we seek to pave the way for MXenes that are engineered from the ground up for biocompatibility, ensuring that these promising 2D materials can transition from the lab bench to biomedical applications safely and sustainably.

## Results

The samples used in this study included Ti_3_C_2_ and Ti_3_C_1.5_N_0.5_, synthesized by selective chemical etching of MAX phase precursors (see Experimental for details). The samples etched using a higher concentration of HCl in the etchant are marked further as Ti_3_C_2_ and Ti_3_C_1.5_N_0.5_. The ones etched in a less concentrated acid are marked as Ti_3_C_2__H_2_O and Ti_3_C_1.5_N_0.5__H_2_O. The samples delaminated using LiCl are identified with Li, and the ones delaminated using NaCl with Na at the end (e.g., Ti_3_C_2__Na and Ti_3_C_1.5_N_0.5__Na). The description of the samples is represented in Table 1.

**Table 1:** Description of the Experimental MXene-Based Samples Used in this Research

MXene	etching	intercalation	encryption
Ti_3_C_2_	HF/HCl	LiCl	Ti_3_C_2__Li
		NaCl	Ti_3_C_2__Na
	HF/HCl/H_2_O	LiCl	Ti_3_C_2__H_2_O_Li
		NaCl	Ti_3_C_2__H_2_O_Na
Ti_3_C_1.5_N_0.5_	HF/HCl	LiCl	Ti_3_C_1.5_N_0.5__Li
		NaCl	Ti_3_C_1.5_N_0.5__Na
	HF/HCl/H_2_O	LiCl	Ti_3_C_1.5_N_0.5__H_2_O_Li
		NaCl	Ti_3_C_1.5_N_0.5__H_2_O_Na

### Scanning Electron Microscopy and Atomic Force Microscopy

In order to characterize the structural and compositional features of the obtained samples, scanning electron microscopy (SEM) with energy dispersive X-ray microanalysis (EDX) and atomic force microscopy (AFM) analysis were performed (Figures 1 and S1–S3). The results of the SEM analysis show that the samples obtained using different etching and exfoliation protocols exhibit a wide variation in the lateral dimensions of the MXenes. However, the average size for all samples is around 1 μm, which correlates well with the DLS results. It can be observed that the MXenes agglomerate into large clusters composed of flakes of various sizes. All samples contain a small concentration of F, Cl, and Na, particularly in those where NaCl was used during the exfoliation process. AFM analysis confirmed the clustered structure of the MXenes. Similar to the SEM results, AFM also revealed that the average lateral size of the MXene flakes is approximately 1 μm, with a broad size distribution. Thickness measurements indicated an average flake thickness of around 4.5 nm. Based on this, we can assume that the synthesized MXenes possess a multilayered structure consisting of at least three layers, considering that the average thickness of a monolayer is approximately 1.5 nm.^21^

**Figure 1:**
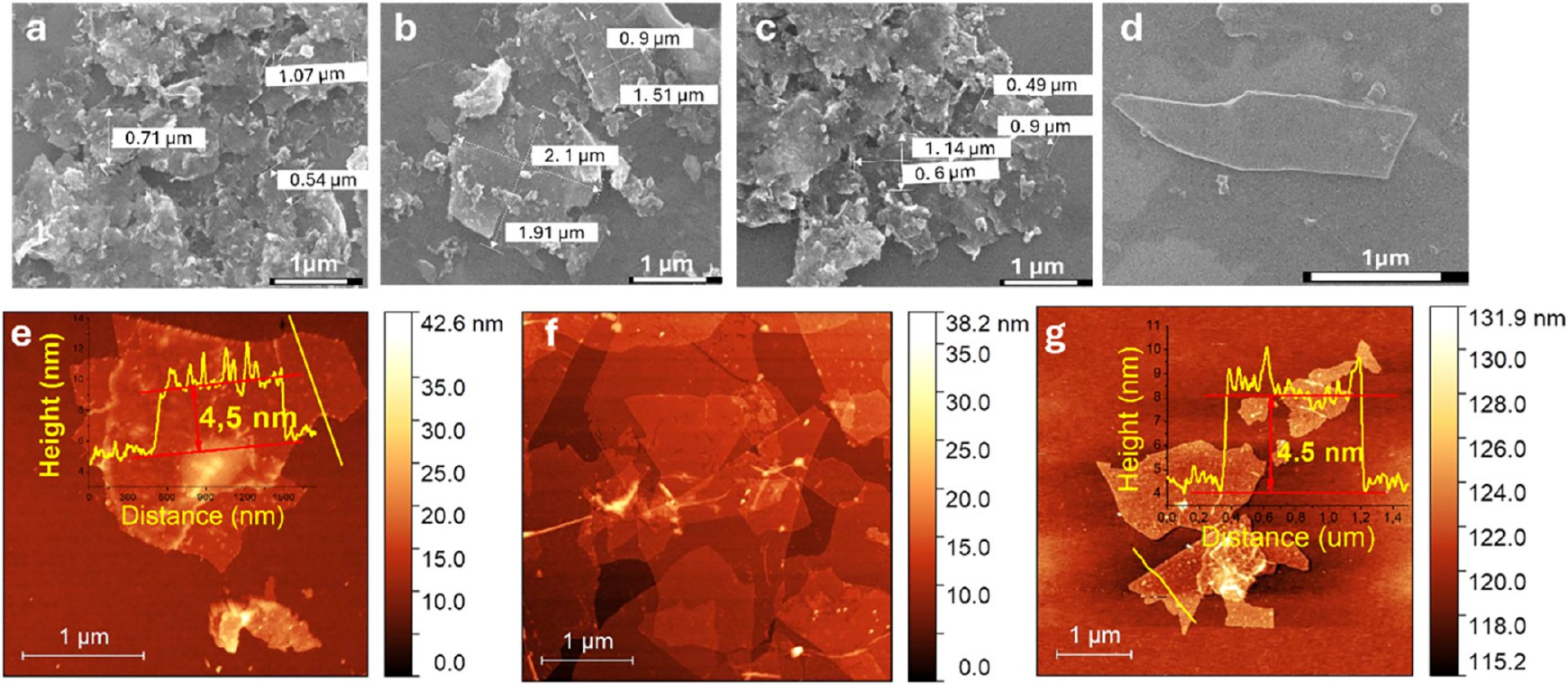
SEM images of Ti_3_C_2_ and Ti_3_C_1.5_N_0.5_ samples, (a, b) Ti_3_C_2__H_2_O_Li; (c) Ti_3_C_1.5_N_0.5__Na, (d) Ti_3_C_1.5_N_0.5__H_2_O_Li; and AFM images of Ti_3_C_2_ and Ti_3_C_1.5_N_0.5_ samples, (e) Ti_3_C_2__H_2_O_Li, (f) Ti_3_C_1.5_N_0.5__Na, (g) Ti_3_C_1.5_N_0.5__H_2_O_Li.

### DLS, UV–vis–NIR, FTIR, and Raman spectroscopy

Flake size and distribution, followed by zeta potential of Ti_3_C_2_ and Ti_3_C_1.5_N_0.5_, are shown in Figure 2. DLS measures particle size based on the assumption that particles are spherical. However, MXenes are flat, 2D flakes, so the size values from DLS may not be precise. These measurements should be viewed as estimates for comparing flake size, not as exact dimensions. However, the trend indicates the difference in size among the produced MXenes. Due to intense delamination by probe sonication, Ti_3_C_2_ and Ti_3_C_1.5_N_0.5_ flakes produced by HCl/HF/H_2_O etching, followed by NaCl intercalation, are reduced in size to submicron dimensions (Figure 2g,h), while the others are in the micrometer range (SI, Table S1). All the suspensions possess high stability as the negative zeta potential is large. In most cases, size reduction of MXene flakes is not desirable, especially in applications where a high electrical conductivity is required. However, it does not play a key role in biomedical applications as long as they are not on a nanoscale (below 100 nm).

**Figure 2:**
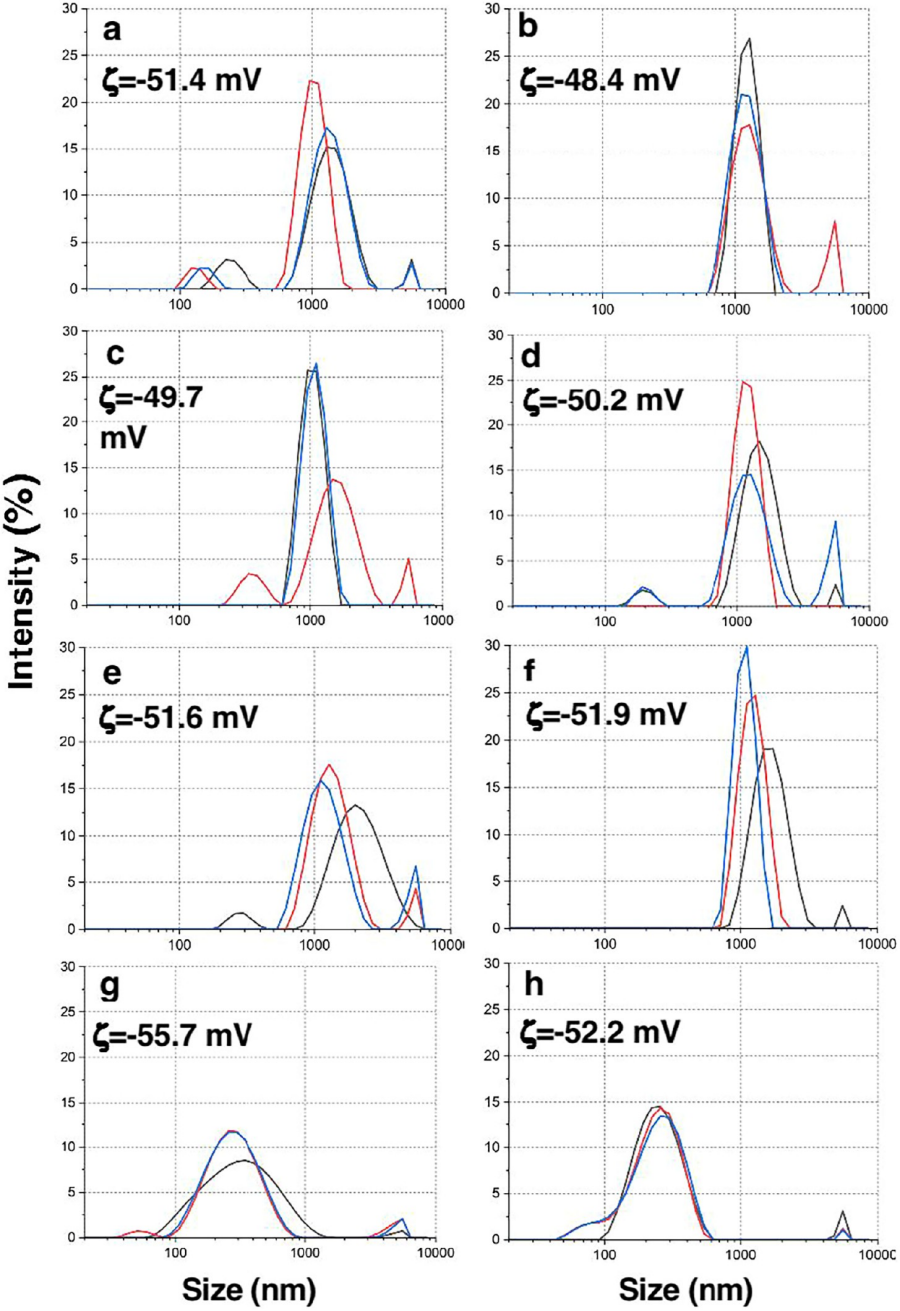
DLS data. (a) Ti_3_C_2__Li, (b) Ti_3_C_1.5_N_0.5_ _Li, (c) Ti_3_C_2__Na, (d) Ti_3_C_1.5_N_0.5__Na, (e) Ti_3_C_2__H_2_O_Li, (f) Ti_3_C_1.5_N_0.5__H_2_O_Li, (g) Ti_3_C_2__H_2_O_Na, (h) Ti_3_C_1.5_N_0.5__H_2_O_Na.

Figure 3 shows the optical properties of produced MXenes, whereas Figure 3a,3b show UV–vis-NIR spectra of Ti_3_C_2_ and Ti_3_C_1.5_N_0.5_, respectively, and Figure 4a shows FTIR spectra. There is no difference in the absorption peak (∼700–800 nm) position between Na^+^ and Li^+^ intercalated Ti_3_C_2_ and Ti_3_C_1.5_N_0.5_ for each etchant (Figure 3a,3b). However, samples etched by a more acidic etchant (HCl/HF) protonate the surfaces more than the diluted one (HCl/HF/H_2_O), and more −OH functional groups at the material surface result in the peaks shifting to lower wavelengths. On the other hand, there is peak splitting in the UV range (interband transitions) and a Ti–O peak appearance at about 250 nm in samples etched with HCl/HF/H_2_O, which is less pronounced in samples etched by HCl/HF (Figure 3a).

**Figure 3:**
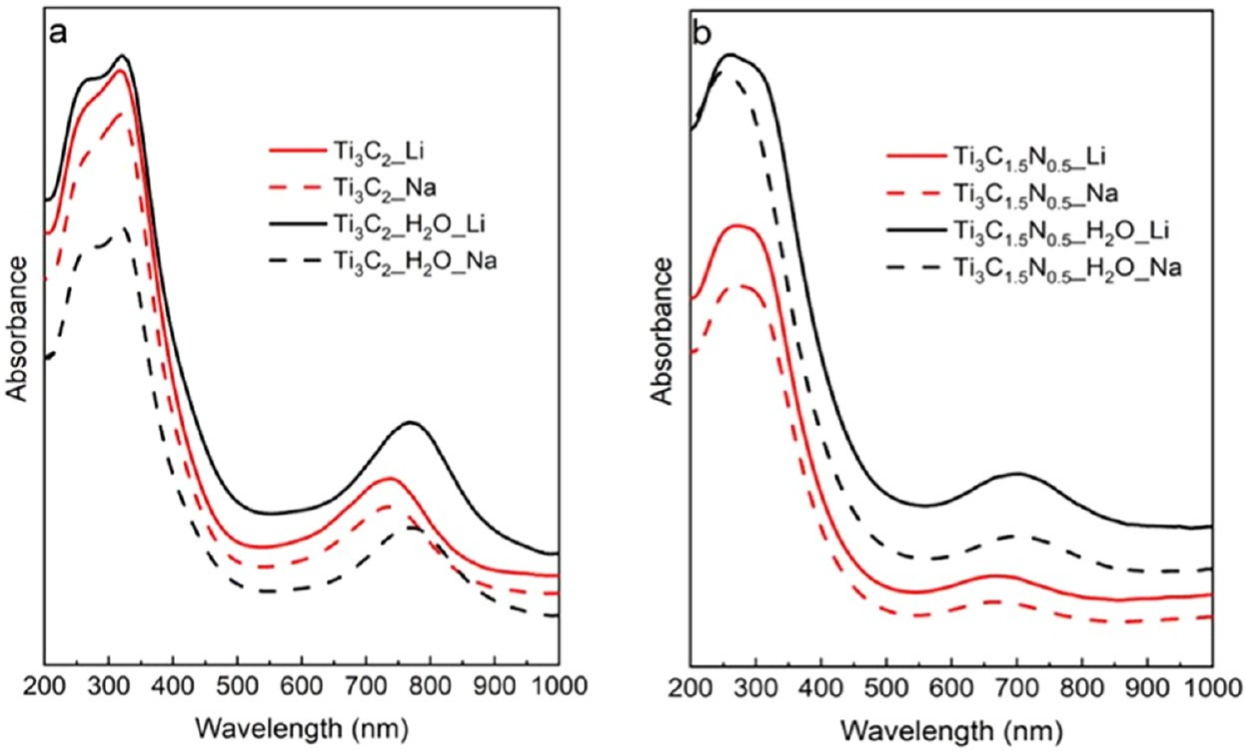
UV–vis-NIR spectra of (a) Ti_3_C_2_ and (b) Ti_3_C_1.5_N_0.5_.

**Figure 4:**
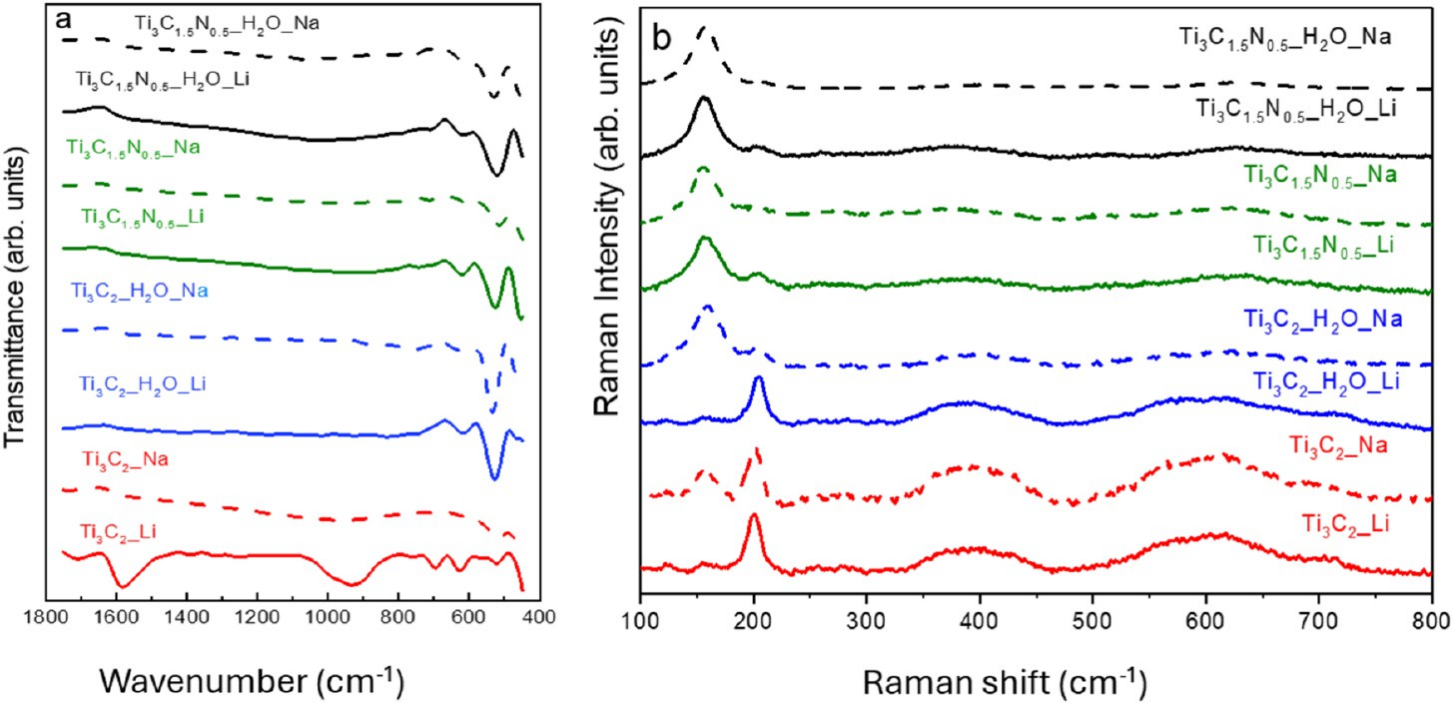
(a) FTIR spectra and (b) Raman spectra of Ti_3_C_2_ and Ti_3_C_1.5_N_0.5_ samples.

The Na^+^ and Li^+^ intercalants do not make a notable impact, and the absorption spectra are only affected by the surface chemistry, T_*x*_, particularly, the –O/–OH ratio. Negligible change in the same wavelength range and less pronounced effects are evident for Ti_3_C_1.5_N_0.5_ (Figure 3b). Figure 4a shows a comparative analysis of FTIR spectra of different MXene samples, demonstrating qualitative differences and similarities in the surface chemistry of all samples.

#### Ti_3_C_2_

The combination of HF/HCl etching and LiCl delamination resulted in the most distinct surface termination peaks. C–O at 1700–1550 cm^–1^ and C–F peaks at 1400–1000 cm^–1^ were dominant for HF/HCl with LiCl, suggesting the presence of residual fluorine and some oxidation. Interestingly, the HF/HCl/H_2_O with LiCl treatment yielded samples with predominant Ti–O peaks (650–550 cm^–1^). This observation requires further investigation, but can also be explained by the fact that Ti_3_C_2_ is prone to oxide formation in aqueous environments. With the introduction of additional H_2_O, we decreased the amount of HF used during etching, which could explain the decrease in fluorine terminations. Additionally, milder etching conditions (presence of H_2_O) might lead to a higher degree of surface hydroxylation (Ti–OH) formation, which could contribute to the observed Ti–O peak intensity.

#### Ti_3_C_1.5_N_0.5_

Compared to Ti_3_C_2_, the differences in surface termination for Ti_3_C_1.5_N_0.5_ under various etching conditions are less pronounced. This is likely due to random positions of N and C atoms in the lattice, leading to peak broadening, and the higher susceptibility of Ti_3_C_1.5_N_0.5_ to oxidation compared with Ti_3_C_2_. Additionally, a broad and indistinguishable C–N peak is observed at 1342–1266 cm^–1^ in all variably etched Ti_3_C_1.5_N_0.5_ samples, unlike the distinct C–F peaks found in Ti_3_C_2_.

To investigate the influence of synthesis conditions on the structural integrity and surface chemistry of Ti_3_C_2_ and Ti_3_C_1.5_N_0.5_ MXenes, Raman spectroscopy was employed as a nondestructive and sensitive technique. Raman spectroscopy revealed distinct structural differences among the MXene samples synthesized via varied etching and intercalation protocols (Figure 4b).

Samples Ti_3_C_2_ and Ti_3_C_2__H_2_O, both intercalated with Li^+^, exhibited nearly identical spectra with a dominant A_1g_ peak at ∼ 201 cm^–1^ and weaker E_g_ modes at 123 and 158 cm^–1^, indicating good structural preservation regardless of water presence in the etching medium.^22–25^ Sample Ti_3_C_2__Na showed a more substantial 155 cm^–1^ mode and a comparable 202 cm^–1^ peak than the Ti_3_C_2__Li sample etched under the same conditions, suggesting that Na^+^ intercalation introduces more significant interlayer distortion. In contrast, Ti_3_C_2_ MXene treated with Na^+^ and H_2_O-assisted etching showed a pronounced shift in spectral features, with the 158–160 cm^–1^ peak becoming dominant and the 202 cm^–1^ A_1g_ mode significantly reduced. Sample Ti_3_C_2__H_2_O_Na also exhibited red-shifted broad bands (∼395 and 620 cm^–1^), indicating enhanced surface disorder and oxidation. The consistent preservation of Raman-active modes in Li^+^-intercalated samples implies a stabilizing effect of lithium on the Ti_3_C_2_ structure. Conversely, Na^+^ intercalation, particularly under aqueous etching, promotes structural asymmetry and degradation.

The Raman spectra of Ti_3_C_1.5_N_0.5_ MXenes samples synthesized under different etching and intercalation conditions reveal pronounced differences in structural features and degree of disorder. All Ti_3_C_1.5_N_0.5_ samples display a dominant peak at ∼ 157 cm^–1^, attributed to in-plane C–Ti–N vibrational modes (E_g_), consistent with previous studies on carbonitride MXenes.^22,26,27^ Ti_3_C_1.5_N_0.5__Li shows a relatively low-intensity 204 cm^–1^ peak (A_1g_, Ti out-of-plane mode) and broad bands at 380 and 615 cm^–1^, indicating moderate structural integrity with some surface oxidation.^28^ In comparison, sample Ti_3_C_1.5_N_0.5__Na presents a more intense 205 cm^–1^ mode and broader bands at 390 and 605 cm^–1^, suggesting that Na^+^ intercalation enhances lattice strain and possibly promotes oxidation. Sample Ti_3_C_1.5_N_0.5__H_2_O_Li exhibits similar spectral features but with reduced 205 cm^–1^ intensity, implying that water-assisted etching introduces additional surface terminations or defect sites, slightly diminishing structural symmetry. Sample Ti_3_C_1.5_N_0.5__H_2_O_Na shows the most degraded profile with a highly intensive 157 cm^–1^ band, a heavily suppressed 205 cm^–1^ peak (5× lower), and weak, broadened features at 390, 510, and 625 cm^–1^, characteristic of advanced disorder and TiO_2_-like oxidation products. The progressive suppression of the A_1g_ mode and emergence of broader bands in this sample indicate cumulative effects of both water in the etching medium and Na^+^ intercalation on structural degradation. Overall, Li^+^-intercalated Ti_3_C_1.5_N_0.5_ samples maintain better-defined vibrational modes, whereas Na^+^ intercalation, particularly under aqueous etching, leads to higher asymmetry, disorder, and potential oxidation.

Comparing both Ti_3_C_2_ and Ti_3_C_1.5_N_0.5_ MXenes, Li^+^-intercalated samples consistently exhibit better preservation of the MXene lattice with more evident A_1g_ modes and reduced disorder. At the same time, Na^+^ intercalation, especially when combined with water-assisted etching, leads to more significant structural distortion, peak broadening, and signatures of surface oxidation. These findings emphasize the importance of synthesis route optimization to preserve the MXene structure and minimize degradation.

### X-ray Photoelectron Spectroscopy (XPS)

A set of XPS spectra recorded for Ti_3_C_2__H_2_O_Li is presented in Figure 5. An analogous set of XPS spectra was acquired for other samples. The survey scan spectrum recorded for Ti_3_C_2__H_2_O_Li, presented in Figure 5a, reveals the presence of fluorine (F 1s signal at ca. 685 eV), oxygen (O 1s signal at ca. 530 eV), titanium (Ti 2p signal at ca. 450 eV), carbon (C 1s signal at ca. 285 eV) and chlorine (Cl 2p signal at ca. 199 eV). Similar signals were also observed for Ti_3_C_1.5_N_0.5_ samples, with an additional characteristic signal from nitrogen −N 1s located at ca. 400 eV (Figure 5a).

**Figure 5:**
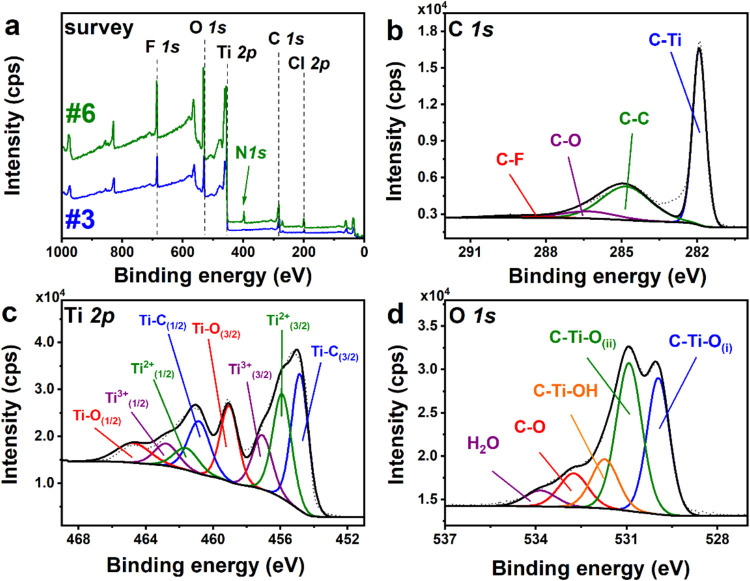
(a) XPS spectra survey spectra recorded for Ti_3_C_2__H_2_O_Li (blue line) and Ti_3_C_1.5_N_0.5__Li (green line), and high-resolution spectra of (b) C 1s, (c) Ti 2p, and (d) O 1s energy regions recorded for Ti_3_C_2__H_2_O_Li.

In the next step, the high-resolution spectra (Figure 5b–5d) were recorded, giving further information about the chemical compositions of the material. The C 1s high-resolution spectrum (Figure 5b) can be deconvoluted into four components with maxima at 281.9, 284.8, 286.3, and 289.2 eV that can be assigned to C–Ti, C–C, C–O, and C–F, respectively.^29–33^ The presence of C–O is probably due to adventitious carbon contaminations.^34^ In the case of Ti_3_C_1.5_N_0.5_, the decomposition of the C 1s spectrum gives similar four components (Figure 6a recorded for Ti_3_C_1.5_N_0.5__Li). However, in this case, the intensity of the component at 286.3 eV is significantly higher due to the contribution of C–N. The presence of C–F bond is also confirmed by analysis of the F 1s high-resolution spectrum (SI, Figure S4a), in which two components can be observed: at 685.5 eV due to intercalated fluoride ions and at 686.8 eV assigned to C–F bond.^30^ On the other hand, chlorine exists only in one form—as intercalated chlorides, as seen from the Cl 2p spectrum recorded for sample Ti_3_C_2__H_2_O_Li (SI, Figure S4b).

**Figure 6:**
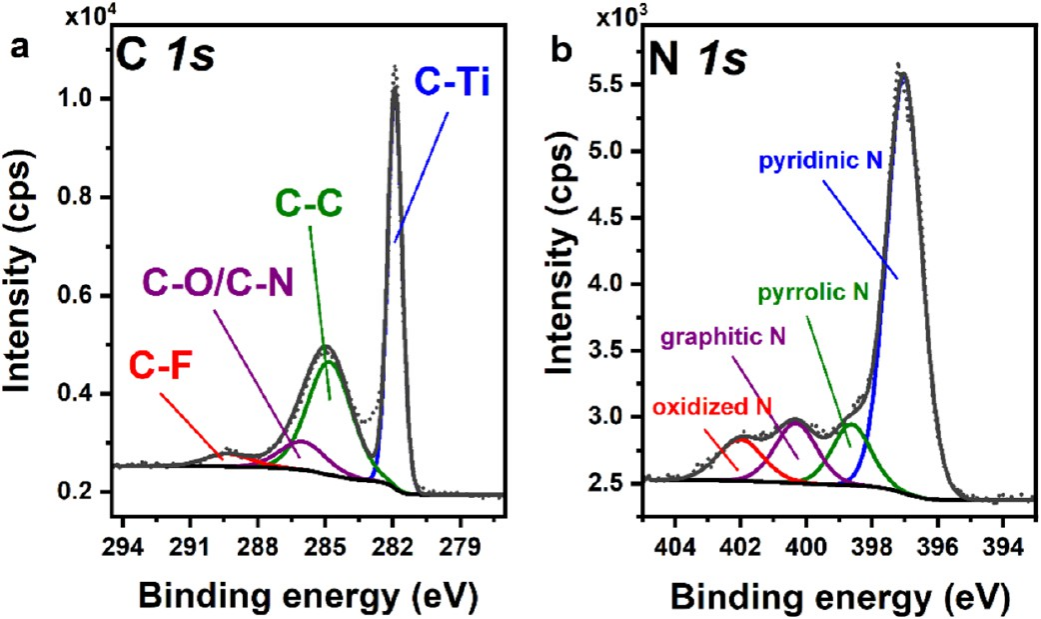
XPS high-resolution spectra of (a) C 1s and (b) N 1s energy regions recorded for the Ti_3_C_1.5_N_0.5__Li sample.

The Ti 2p high-resolution spectrum (Figure 5c) comprises four components (2p_3/2_), with their spin–orbit splitting counterparts (2p_1/2_), that are located at 454.8 eV (Ti–C), 455.9 eV (Ti^2+^), 457.1 eV (Ti^3+^), and 459.0 eV (Ti–O). No Ti 2p_3/2_ component was observed at 460 eV—previously reported for Ti–F bond, thus, direct attachment of F atoms to Ti is excluded. The analysis of the Ti 2p spectra recorded for other samples reveals the presence of the same components with varied ratios. The deconvolution of the O 1s spectrum (Figure 5d) gives five components: C–Ti–O at 529.9 and 530.9 eV, C–Ti–OH at 531.7 eV, C–O at 532.7 eV, and an additional component at 533.8 eV due to strongly bonded water molecules.^29,30^

For Ti_3_C_1.5_N_0.5,_ an additional N 1s high-resolution spectrum was acquired. The exemplary spectrum, recorded for Ti_3_C_1.5_N_0.5__Li, is shown in Figure 6b. The analysis gives four components with maxima at 397.8, 399.4, 401.1, and 402.8 eV, that can be assigned to pyridinic, pyrrolic, graphitic, and oxidized nitrogen, respectively.^35^

### Biocompatibility

The cytotoxicity assays and cell-death analyses (Figures 7–9) reveal clear trends in how MXene composition and synthesis affect biocompatibility. Across all samples and time points, HaCaT cell viability was strongly dose-dependent: exposure to low MXene doses (6.25 μg/mL) caused minimal or lack of toxicity, whereas higher doses (50–100 μg/mL) progressively reduced cell viability. In particular, the resazurin reduction assays over 6 days (Figure 7) showed that, even at the longest exposure, 6.25 μg/mL of MXene maintained viability comparable to untreated controls, while 100 μg/mL treatments caused a significant drop in metabolic activity and cell proliferation (*p* < 0.05 vs control). Intermediate concentrations (12.5–50 μg/mL) produced moderate effects, indicating a threshold-like behavior where cytotoxicity markedly accelerates at the upper end of the tested dose range.^36^ These observations are consistent with literature reports that Ti_3_C_2_T_*x*_ MXenes generally sustain >70–80% cell viability at ≤ 50 μg/mL, with more pronounced toxicity only emerging at higher doses or prolonged exposure.^37^

**Figure 7:**
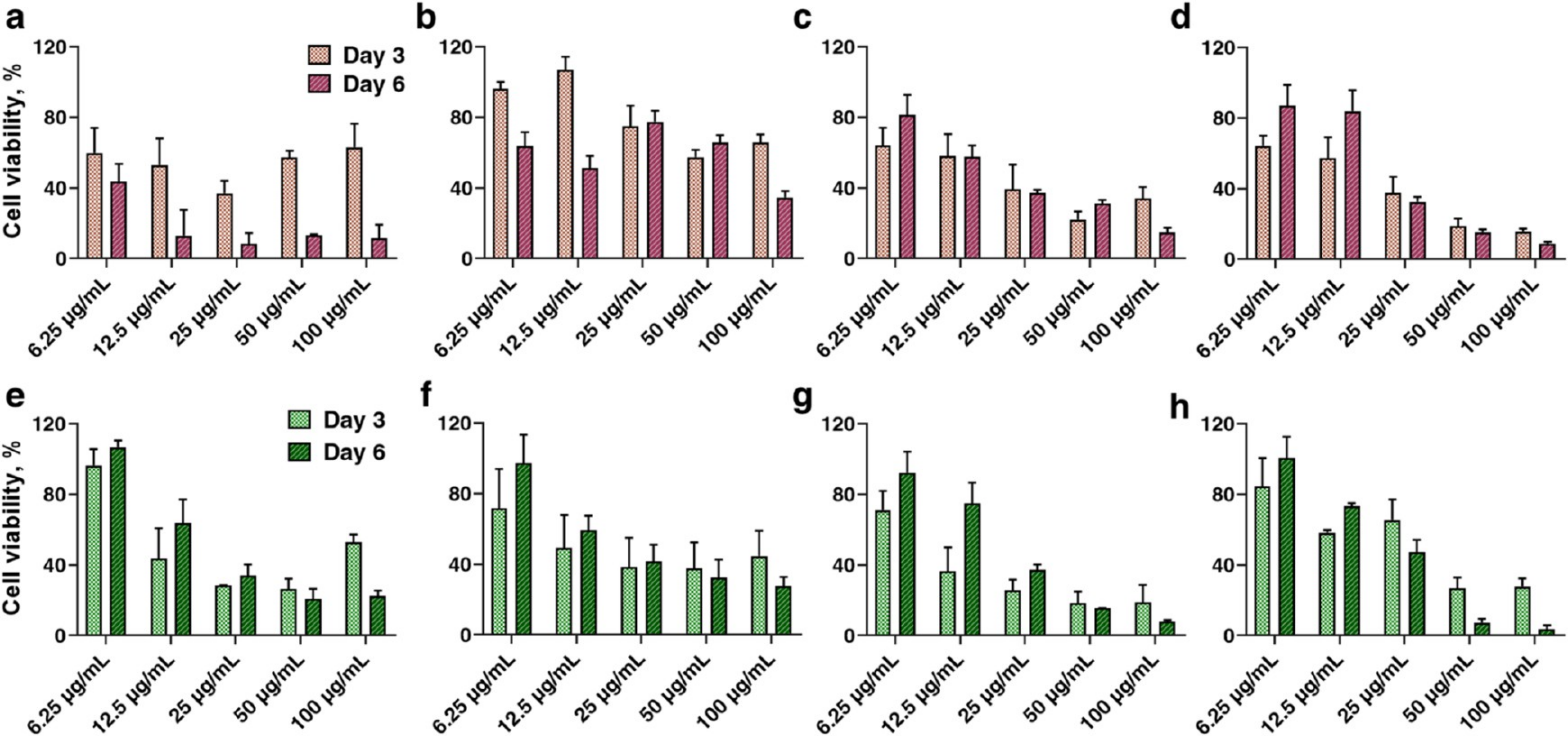
Dose- and synthesis-dependent cytotoxicity of Ti_3_C_2_ and Ti_3_C_1.5_N_0.5_ MXenes assessed by resazurin reduction assay over 6 days in HaCaT keratinocytes (*n* = 3), where (a) Ti_3_C_2__Li, (b) Ti_3_C_2__Na, (c) Ti_3_C_2__H_2_O_Li, (d) Ti_3_C_2__H_2_O_Na, (e) Ti_3_C_1.5_N_0.5__Li, (f) Ti_3_C_1.5_N_0.5__Na, (g) Ti_3_C_1.5_N_0.5__H_2_O_Li, and (h) Ti_3_C_1.5_N_0.5__H_2_O_Na.

The cytotoxic outcome of MXene strongly depends on its composition (carbide vs carbonitride). Ti_3_C_1.5_N_0.5_ MXene was considerably more biocompatible than Ti_3_C_2_ ones under equivalent conditions. After 6 days at the highest concentration, Ti_3_C_1.5_N_0.5_-treated cells retained substantially higher viability compared to Ti_3_C_2_-treated cells (Figure 7). Even at intermediate doses (25–50 μg/mL), Ti_3_C_1.5_N_0.5_ caused only a partial reduction in resazurin signal, whereas Ti_3_C_2_ often induced a stronger viability loss. This suggests that partial substitution of carbon with nitrogen in the MXene structure yields a more biofriendly surface chemistry or dissolution profile. Indeed, our surface analyses showed that Ti_3_C_1.5_N_0.5_ surfaces carry fewer—F terminations and are more oxidized/hydroxylated than Ti_3_C_2_, which may inherently mitigate toxicity. In line with this, all Ti_3_C_1.5_N_0.5_ samples maintained cell viabilities above ∼ 80% at 25 μg/mL and showed no significant differences between etching routes, indicating a robust and relatively inert behavior in the biological medium.

In contrast, Ti_3_C_2_ MXene’s cytotoxicity was highly sensitive to synthesis parameters. The etching route, in particular, had a notable effect: including an H_2_O step in the etchant (HF/HCl/H_2_O) markedly improved Ti_3_C_2_ biocompatibility relative to the conventional HF/HCl etch. By day 6, Ti_3_C_2_ produced via HF/HCl/H_2_O etching showed significantly higher cell viability than its HF/HCl-etched at the same doses (especially at 50–100 μg/mL). In fact, HF/HCl/H_2_O-etched Ti_3_C_2_ behaved almost as the Ti_3_C_1.5_N_0.5_ MXenes, causing only mild (∼10–20%) viability reduction at 25 μg/mL over 6 days. Without the water-assisted etching, however, Ti_3_C_2_T_*x*_ was substantially more toxic: the HF/HCl-only etched Ti_3_C_2_ caused a pronounced viability drop even at 25 and 100 μg/mL it led to a near-complete reduction of cell growth (Figure 7). This dramatic difference correlates with the MXene’s surface termination profiles— water-assisted etching reduced surface fluorination and enriched −OH terminations, whereas the HF/HCl route left abundant −F groups on Ti_3_C_2_T_*x*_. Fluoride termination is known^38^ to render MXenes less hydrophilic and can introduce cytotoxic effects (e.g., due to release of F^–^/HF or unfavorable cell membrane interactions). Conversely, −OH terminations improve hydrophilicity and are generally considered^9^ much cell-friendly. Our findings reinforce this: the F-rich Ti_3_C_2_ sample (HF/HCl etch) was the most cytotoxic, while the −OH-rich Ti_3_C_2_ (HF/HCl/H_2_O etch) was significantly more biocompatible.

The intercalating cation (Li^+^ vs Na^+^) also emerged as an important factor for Ti_3_C_2_. Na^+^-intercalated Ti_3_C_2_ consistently outperformed its Li-intercalated equivalent in biocompatibility assays. For example, Ti_3_C_2_ etched with HF/HCl/H_2_O and intercalated with Na^+^ induced minimal cytotoxicity, with cell viability >90% at 25 μg/mL (24 h exposure) and only ∼ 20% loss at 100 μg/mL, whereas the same MXene etched identically but intercalated with Li^+^ caused notably greater cell death. Similarly, under the harsher HF/HCl etch, replacing Li^+^ with Na^+^ during delamination led to a modest but reproducible improvement in viability (Figure 7). The most toxic formulation overall was Ti_3_C_2__Li, which caused the most significant reduction in HaCaT viability over time. By contrast, Ti_3_C_2__H_2_O_Na was the least toxic Ti_3_C_2_ sample, approaching the low cytotoxicity of Ti_3_C_1.5_N_0.5_. These trends align with recent insights that residual Li on MXene surfaces can contribute to cytotoxicity.^3^ If not completely removed, Li^+^ ions or Li-based impurities (from LiF/LiCl) may leach out and disturb cellular ion homeostasis. Na^+^, being larger and more easily washed out, or forming a thicker hydration shell on MXene layers, leads to an increase in biocompatibility. We indeed observed that all Li^+^-intercalated Ti_3_C_2_ samples triggered slightly higher levels of cell stress (e.g., a trend toward increased IL-8 cytokine release) compared to Na-intercalated ones, whereas Ti_3_C_1.5_N_0.5_ showed negligible intercalant-related differences.

Apart from terminations, particle size and dispersibility are additional key factors linking synthesis to bioperformance. DLS measurements (Figure 3) showed that the HF/HCl/H_2_O + Na^+^ protocol yielded smaller, submicron MXene flakes (∼280 nm lateral size), whereas the HF/HCl + Li^+^ route preserved larger, ∼ 1–2 μm flakes (with other variants in between). All dispersions had strongly negative zeta potentials, but the smaller Na-intercalated flakes formed more stable colloids with less restacking. Biologically, this size difference likely contributes to the observed reduced cytotoxicity of the Na-intercalated samples. Smaller monolayer flakes can more easily remain suspended and evenly distributed, preventing large agglomerates from settling onto cells. In contrast, larger MXene stacks may sediment and physically cover cell surfaces, causing localized high doses and mechanical strain on the cell membrane.

To examine cell-death mechanisms underlying the viability trends, we conducted flow cytometry (apoptosis and necrosis assays) after 24 h exposure at a representative dose of 25 μg/mL (Figure 8). The annexin V/PI staining results support the viability data: Ti_3_C_1.5_N_0.5_ MXenes caused almost no apoptosis or necrosis at 25 μg/mL, with death rates comparable to control cells. For Ti_3_C_2_, water-etched/Na-intercalated samples induced very low levels of cell death (only a small percentage of annexin V-positive early apoptotic cells, and virtually no PI-positive necrotic cells). In contrast, the HF/HCl-etched, Li^+^-intercalated Ti_3_C_2_ led a much higher fraction of cells into apoptosis/necrosis. Specifically, Ti_3_C_2__Li exposure resulted in a large annexin V–positive population and appreciable PI uptake, indicating that a significant subset of cells underwent programmed cell death or lytic death within 24 h. Quantitatively, this sample exhibited the highest combined apoptotic+necrotic percentage among all conditions. Other Ti_3_C_2_ variants fell in between: for example, HF/HCl-etched Na^+^-MXene and HF/HCl/H_2_O-etched Li^+^-MXene induced moderate levels of annexin V positivity but low primary necrosis. Notably, in most cases, apoptosis was more prevalent than necrosis at 25 μg/mL, suggesting that MXene-induced cell death at sublethal doses occurs largely via programmed apoptotic pathways rather than immediate membrane rupture. This is consistent with the idea that MXene exposure generates oxidative or metabolic stress that triggers apoptosis, as opposed to outright necrotic injury. Indeed, prior studies have linked MXene-induced ROS generation to mitochondrial dysfunction and downstream apoptotic signaling in cells.

**Figure 8:**
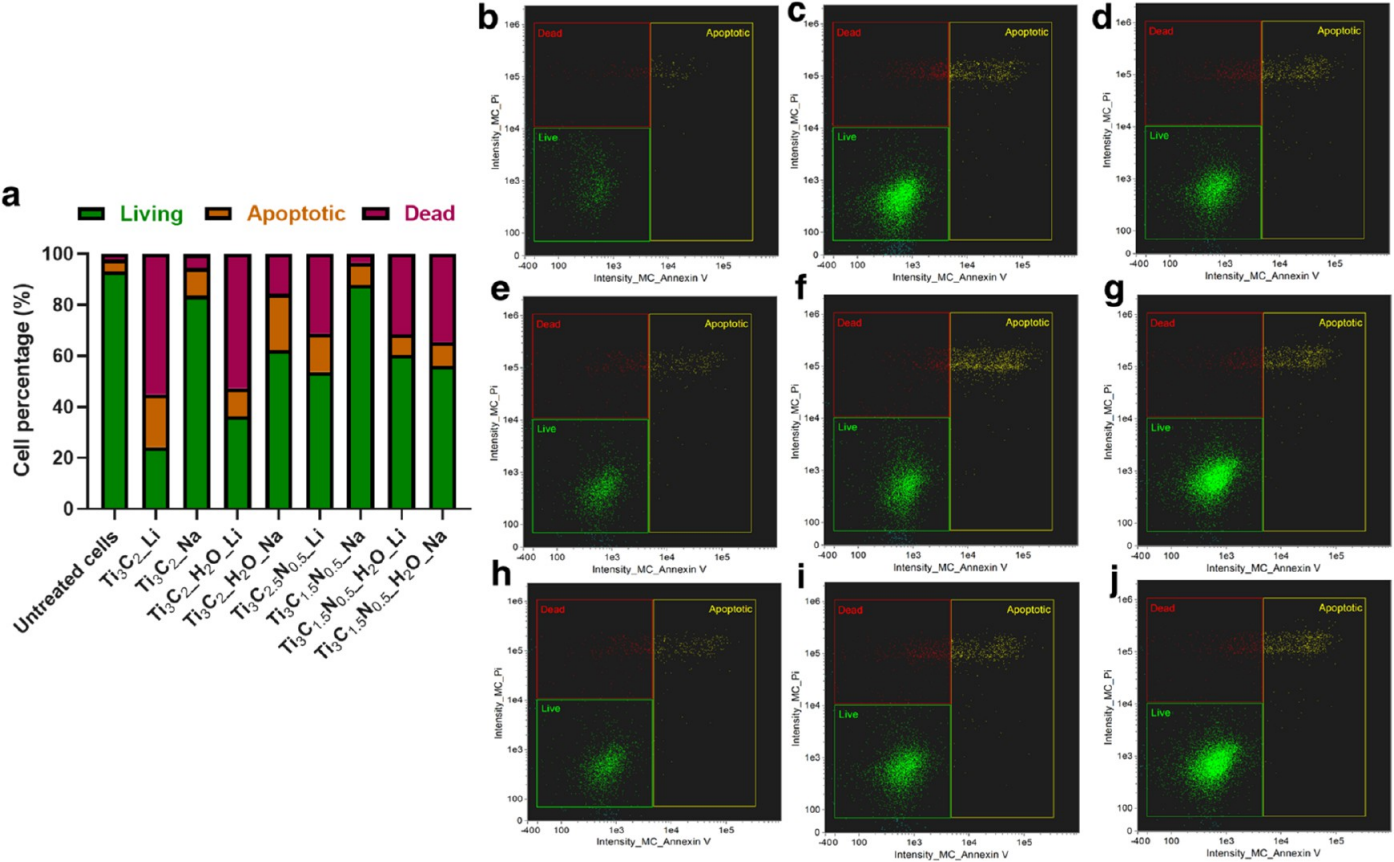
Flow cytometric assessment of apoptosis and necrosis in HaCaT cells after 24 h exposure to 25 μg/mL of various MXenes (a) and representative dot plots of Annexin V vs PI staining (*n* = 3), where (b) untreated cells, (c) Ti_3_C_2__H_2_O_Li, (d) Ti_3_C_1.5_N_0.5__Na, (e) Ti_3_C_2__Li, (f) Ti_3_C_2__H_2_O_Na, (g) Ti_3_C_1.5_N_0.5__H_2_O_Li, (h) Ti_3_C_2__Na, (i) Ti_3_C_1.5_N_0.5__Li and (j) Ti_3_C_1.5_N_0.5__H_2_O_Na.

The live/dead fluorescence imaging (Figure 9) provides a visual confirmation of these dose-dependent and sample-dependent effects. HaCaT cultures treated with 6.25 μg/mL of any MXene showed uniform green fluorescence (Calcein AM–stained live cells) with almost no red nuclei (PI–stained dead cells), similar to untreated controls. At 25 μg/mL, cell monolayers remained largely intact and green, but isolated red-stained cells appeared, indicating that a fraction of cells had lost viability. Importantly, the density of dead (red) cells at 25 μg/mL correlated with MXene type: fields treated with the most biocompatible samples (e.g., Ti_3_C_2__H_2_O_Na) showed only the occasional red cell, whereas those treated with the more toxic Ti_3_C_2__Li had noticeably more red cells among the green, reflecting its higher apoptotic/necrotic rate. At the highest dose of 100 μg/mL, extensive cell death was observed for all MXenes— the fluorescence images revealed largely red fluorescing nuclei and cell debris, with very few viable cells remaining. This dramatic shift at 100 μg/mL underscores the strong membrane-disrupting potential of high-concentration MXenes, as evidenced by widespread PI penetration into cells. The data suggest that at such a high burden, MXene flakes can overwhelm cellular defenses and cause acute membrane rupture and necrosis. Two-dimensional flakes at high density may physically perturb lipid bilayers, especially if agglomerated or if bearing less hydrophilic surface groups. Oxidative stress may further exacerbate this damage, compounding the loss of membrane integrity. By contrast, at sublethal doses (≤25 μg/mL), cell membranes remained largely intact and cell death was limited, in agreement with the flow cytometry showing mainly early apoptotic changes without bulk necrosis. The live/dead assay thus illustrates the dose threshold between biocompatible and cytotoxic levels of MXenes, while also mirroring the relative cytotoxic rankings of the different MXene formulations.

**Figure 9:**
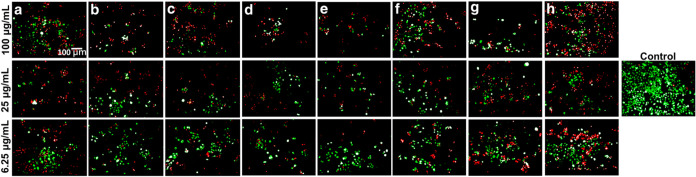
Fluorescence live/dead staining of HaCaT cells after 24 h incubation with Ti_3_C_2_ and Ti_3_C_1.5_N_0.5_ MXenes at concentrations of 6.25, 25, and 100 μg/mL. (a) Ti_3_C_2__Li, (b) Ti_3_C_2__Na, (c) Ti_3_C_2__H_2_O_Li, (d) Ti_3_C_2__H_2_O_Na, (e) Ti_3_C_1.5_N_0.5__Li, (f) Ti_3_C_1.5_N_0.5__Na, (g) Ti_3_C_1.5_N_0.5__H_2_O_Li, and (h) Ti_3_C_1.5_N_0.5__H_2_O_Na. The magnification of the images is ×10 (scale bar = 100 μm).

Surface chemistry analyses (FTIR, XPS) of MXenes help explain the biological trends. As noted, HF/HCl etching yields Ti_3_C_2_T_*x*_ with considerable surface fluorination, whereas adding water during etching reduces −F and increases −O/–OH terminations. XPS confirmed that Ti_3_C_2__Li had prominent C–F and low O–Ti/O–C signals, while Ti_3_C_2__H_2_O samples showed the opposite, with dominant O–Ti peaks indicating a more oxygen-terminated surface. These chemical differences correlate directly with cytotoxicity: the F-terminated Ti_3_C_2_ likely leaches more fluoride or other reactive species upon slight oxidation (e.g., Ti–F bonds can hydrolyze, releasing F^–^), and is less hydrophilic, which can disrupt cell membranes and increase oxidative stress. On the other hand, the OH-terminated Ti_3_C_2_ is more stable in aqueous environments and interacts more gently with cells, leading to much lower ROS generation and membrane perturbation. Similarly, Ti_3_C_1.5_N_0.5_ was found to be inherently less fluorinated—in all synthesis variants, its surfaces had mostly O/N functional groups and very little detectable F. This absence of surface fluorine is a likely reason why all Ti_3_C_1.5_N_0.5_ samples were relatively noncytotoxic. In essence, surface terminations act as the “chemical interface” to cells: more bioinert groups (–O, −OH) promote compatibility, while electronegative or less stable groups (–F, =O to some extent) can trigger stress responses. Our results strongly support this paradigm. They are also in agreement with recent reports that eliminating halogen terminations yields extremely biocompatible MXenes. For example, Yoon et al. found that Ti_3_C_2_T_*x*_ synthesized via halogen-free NaOH etching caused no significant cytotoxicity even at 2 mg/mL, whereas a conventional HF-etched Ti_3_C_2_T_*x*_ caused ∼ 50% cell viability loss at the same dose.^9^ We demonstrate that even within partially fluorinated MXenes, incremental reduction of −F (through modified etching) translates to appreciable gains in biocompatibility. It should be noted that these results warrant further validation in other cell types, including cancer models, as several studies have shown that cancer cells may exhibit heightened sensitivity to MXenes due to their altered redox states and membrane vulnerabilities.^16^

### Toxicity Mechanisms: Oxidative Stress and Inflammatory Response

To assess oxidative stress, intracellular ROS generation was quantified in HaCaT keratinocytes exposed to Ti_3_C_2_T_*x*_ and Ti_3_C_1.5_N_0.5_T_*x*_ MXenes (6.25, 25, 100 μg/mL) over 2, 4, and 6 h (Figure 10). Distinct ROS kinetic profiles emerged depending on MXene composition and synthesis. At the highest dose (100 μg/mL), Ti_3_C_1.5_N_0.5_T_*x*_ elicited a relatively delayed oxidative response, with ROS levels peaking only at ∼ 6 h postexposure across all etching/intercalation variants. In contrast, Ti_3_C_2_T_*x*_ induced more rapid ROS bursts: for example, Na-intercalated Ti_3_C_2_T_*x*_ reached maximal ROS as early as 2 h, whereas Li-intercalated Ti_3_C_2_T_*x*_ peaked at ∼ 4 h. This indicates that the MXene’s surface chemistry (depending on intercalant type and etching route) modulates the timing of oxidative stress. Interestingly, at lower, subcytotoxic concentrations (25 and 6.25 μg/mL), the peak ROS generation shifted to 6 h for all MXenes, suggesting that higher doses trigger acute bursts of ROS while lower doses lead to a slower, sustained ROS accumulation. In terms of magnitude, intermediate MXene doses tended to evoke the most pronounced ROS elevations relative to control, whereas at 100 μg/mL, some formulations showed attenuated ROS response (possibly due to overt cytotoxicity activity). Notably, among all formulations, the water-etched, Na-intercalated MXenes yielded the lowest ROS levels—Ti_3_C_2_T_*x*_ produced via HF/HCl/H_2_O etching with Na^+^ showed no significant ROS increase versus untreated cells at these time points. This suggests that incorporating an H_2_O step (promoting −OH terminations) and Na^+^ ions during synthesis yields MXenes with a markedly reduced oxidative footprint. In contrast, MXenes produced by the H_2_O-free (HF/HCl) route and/or intercalated with Li^+^ tended to generate higher ROS, implying that residual fluorinated terminations or Li-related species intensify oxidative stress.

**Figure 10:**
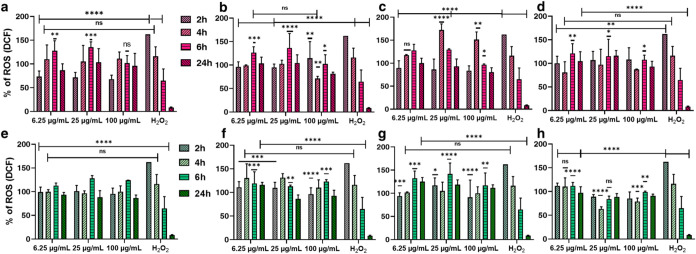
ROS generation potential of different types of MXenes. (a) Ti_3_C_2__Li, (b) Ti_3_C_2__Na, (c) Ti_3_C_2__H_2_O_Li, (d) Ti_3_C_2__H_2_O_Na, (e) Ti_3_C_1.5_N_0.5__Li, (f) Ti_3_C_1.5_N_0.5__Na, (g) Ti_3_C_1.5_N_0.5__H_2_O_Li and (h) Ti_3_C_1.5_N_0.5__H_2_O_Na. All statistical significance indicated difference with positive control (treated by H_2_O_2_ 3 mM): *p ≤ 0.05, **p ≤ 0.01, ***p ≤ 0.001, ****p ≤ 0.0001, and ns = nonsignificant (*p* ≥ 0.05). Error bars represent mean ± SD from three independent experiments, (*n* = 3).

These ROS dynamics highlight how compositional and surface differences translate to cellular oxidative responses. Ti_3_C_2_ versus Ti_3_C_1.5_N_0.5_ MXenes showed divergent behavior: the carbonitride generally induced lower and later ROS peaks than the carbide, aligning with its overall milder cytotoxicity. However, elemental composition alone was not the decisive factor—surface functionalization and intercalation chemistry played a more critical role than the M/X stoichiometry. For instance, Ti_3_C_1.5_N_0.5_T_*x*_ synthesized via HF/HCl/H_2_O showed minimal ROS unless intercalated with Li^+^ (in which case, ROS levels rose significantly compared to the Na-intercalated analog). Likewise, switching the Ti_3_C_2_T_*x*_ etching route from HF/HCl to HF/HCl/H_2_O (thereby enriching −OH/–O terminations and reducing −F) dramatically dampened its ROS generation. The presence of surface −OH groups is known to mitigate oxidative reactions,^39^ whereas heavily fluorinated surfaces (from HF etching without water) correlate with greater ROS release, likely because −F terminations make the MXene more redox-active or leave acidic/oxidizing residues. These trends are in agreement with reports that conventional HF-etched (F-terminated) Ti_3_C_2_T_*x*_ can induce significant ROS stress in cells compared to MXenes with fewer −F terminations. Excess surface fluoride or residual intercalant cations (e.g., Li^+^) have indeed been implicated in provoking inflammation and cytotoxicity.^40^ By contrast, MXenes engineered with predominantly O/OH terminations can even exhibit antioxidant behavior—for example, Zhao et al. found that Ti_3_C_2_T_*x*_ nanosheets effectively scavenged ROS and protected chondrocytes from oxidative injury in vitro.^41^ Consistent with this, a recent in vivo study demonstrated that the intrinsic oxidative potential of Ti_3_C_2_T_*x*_ is a key determinant of its inflammatory toxicity, as MXene-induced lung inflammation in mice was greatly abated by cotreatment with an ROS scavenger (*N*-acetylcysteine).^40^ Taken together, our results and the literature concur that controlling MXene surface terminations (−F vs −OH) and residual species is crucial for modulating oxidative stress outcomes.

Inflammatory signaling in MXene-exposed keratinocytes was assessed by measuring secreted cytokine levels (Figure 11). IL-6, a major pro-inflammatory cytokine, remained unchanged across all MXene treatments (25 μg/mL, 24 h), with no significant increase relative to control cells. This uniform lack of IL-6 elevation indicates that none of the MXene formulations triggered an overt acute inflammatory response or “cytokine storm” in keratinocytes. Even samples that caused considerable ROS did not elicit IL-6 secretion, underscoring that acute MXene exposure (at moderate doses) does not strongly activate IL-6-mediated pathways associated with inflammation. Ti_3_C_1.5_N_0.5_T_*x*_ in particular showed a slight trend toward lower IL-6 release than Ti_3_C_2_T_*x*_, again reflecting its more favorable interaction with cells. In contrast to IL-6, IL-8 levels were significantly modulated by MXene exposure in a size- and chemistry-dependent manner. IL-8 is a chemokine that recruits neutrophils and is often upregulated in response to cellular stress. The smallest MXene flakes (lateral size ≤ 280 nm) actually reduced IL-8 secretion compared to the untreated control. For example, the Ti_3_C_2_T_*x*_ sample produced by HF/HCl/H_2_O etching with Na^+^ (average ∼ 280 nm flake size) showed the lowest IL-8 levels, on par with or below baseline, hinting at a mild anti-inflammatory or immunosuppressive effect by these nanoscale sheets. Similarly, the Ti_3_C_1.5_N_0.5__Na sample (also sub-300 nm) induced no significant IL-8 increase. In contrast, MXene formulations with larger lateral dimensions (∼1.3 μm and above) provoked an elevated IL-8 response. All samples composed of large flakes elicited significantly higher IL-8 secretion than control cells, consistent with a pro-inflammatory reaction to the bigger particulate stimuli. Thus, a clear relationship was observed between MXene flake size and IL-8 release: smaller, well-dispersed flakes tend to dampen IL-8 (or at least not stimulate it), whereas larger particles drive IL-8 upregulation. This size-dependent immunological effect aligns with reports on MXene quantum dots (ultrasmall Ti_3_C_2_ fragments), which have been shown to suppress IL-6 and IL-8 production in endothelial cells.^42^ They found that Ti_3_C_2_ QDs, despite causing some cytotoxicity at high doses, did not promote IL-6/IL-8 secretion—rather, these QDs significantly reduced baseline IL-6/IL-8 levels. Our findings mirror this behavior at the upper end of the nanosize spectrum, suggesting that when MXene sheets are sufficiently small, they may evade or even dampen certain inflammatory pathways. On the other hand, larger micron-sized flakes likely activate pattern recognition receptors or cause persistent stress that leads to IL-8 release from keratinocytes (a common response to foreign particulates).

**Figure 11:**
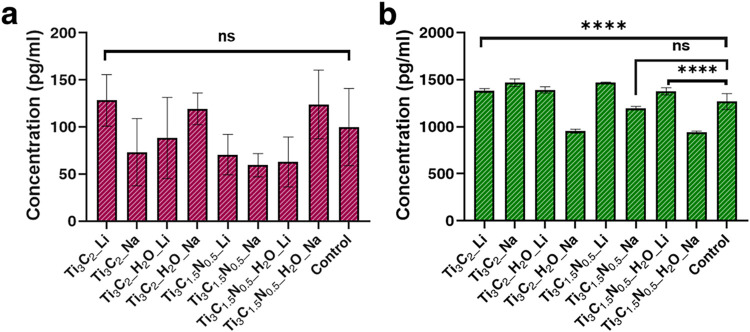
Quantification of (a) IL-6 and (b) IL-8 secretion in HaCaT cells treated with different MXene formulations. All statistical significance indicated a difference with control: ****p ≤ 0.0001 and ns = nonsignificant (*p* ≥ 0.05). Error bars represent mean ± SD from three independent experiments, (*n* = 3).

Beyond size, surface chemistry, and intercalant identity also influenced cytokine profiles. The lowest IL-8 levels were consistently observed for MXenes synthesized with the H_2_O-containing etch and Na^+^ intercalation—the same samples that showed minimal ROS generation. This correlation implicates surface terminations (and associated residual species) in the inflammatory outcome. We noted that Ti_3_C_1.5_N_0.5_T_*x*_ samples intercalated with Li^+^ induced higher IL-8 secretion than their Na-intercalated counterparts, whereas for Ti_3_C_2_T_*x*_, he intercalant made less difference (IL-8 was driven more by flake size and etching method). One interpretation is that Li-intercalated MXenes carry trace Li compounds or distinct terminations that can stimulate inflammatory signaling in cells, an effect more pronounced in the Ti_3_C_1.5_N_0.5_ composition. In Ti_3_C_2_T_*x*_, the strong size effect (and possibly a universally high surface reactivity) may mask any subtle Li vs Na differences on IL-8. Overall, the trends in IL-8 mirror the ROS results: the formulations that caused an early, transient ROS peak (e.g., small, O-terminated Na^+^-MXenes) elicited negligible IL-8, whereas those with delayed or sustained ROS (e.g., larger, F-terminated flakes) corresponded to significant IL-8 elevation by 24 h. This suggests that persistent oxidative stress is a key trigger for IL-8 production. Prolonged ROS can activate redox-sensitive transcription factors (NF-κB, AP-1) that drives IL-8 expression, linking the oxidative stress response to downstream chemokine release. Indeed, in the broader context of 2D materials, inflammation has been shown to closely track the material’s “oxidative potential”.^40^ Our data support this hypothesis: MXenes with higher intrinsic ROS-generating capacity promote a pro-inflammatory IL-8 response, whereas those engineered to minimize ROS can avoid or even counteract inflammatory signaling.

In general, the oxidative stress and inflammatory assessments illustrate a coherent synthesis-to-response relationship (SI, Table S3). MXene compositions and processing that yield smaller flakes with OH-/O-terminated surfaces (minimal −F, no excess Li^+^) were the most biocompatible in terms of oxidative and inflammatory outcomes, causing only transient or low ROS and negligible IL-6/IL-8 release (even exhibiting antioxidative/anti-inflammatory tendencies). In contrast, MXenes with more conventional HF-derived surfaces (abundant −F terminations, less OH) and larger lateral size induced higher ROS levels and a notable IL-8 chemokine response, hallmarks of cellular oxidative stress and pro-inflammatory activation. These findings underscore the importance of tailoring MXene surface chemistry via etching and intercalation. By reducing surface fluorination and carefully choosing intercalant (e.g., using Na^+^ with water-mediated exfoliation), it is possible to attenuate MXene-induced ROS generation and thereby mitigate inflammation. Conversely, leaving aggressive surface terminations or residual etchants can exacerbate oxidative stress, which in turn drives inflammatory signaling. This mechanistic understanding aligns with existing data on MXene toxicity, reinforcing that the “oxidative stress paradigm” is central to MXene–cell interactions and that strategic surface modifications can tip the balance toward either pro-oxidant, inflammatory outcomes or a more benign, even protective, biological profile.^41^ Such insights are valuable for guiding the design of MXenes that are safer for biomedical applications, ensuring that these 2D nanomaterials can be harnessed with minimal disruption to cellular redox homeostasis and immune parameters.

Additionally, the consistently higher biocompatibility of Ti_3_C_1.5_N_0.5_T_*x*_ relative to Ti_3_C_2_T_*x*_ can be attributed to synergistic differences in surface chemistry, redox activity, and structural stability imparted by nitrogen incorporation. First, Ti_3_C_1.5_N_0.5_T_*x*_ possesses a more cell-friendly surface termination profile. Water-assisted etching with Na^+^ yielded carbonitride flakes with minimal −F groups and abundant −OH/–O terminations, thereby enhancing hydrophilicity and removing a key source of cytotoxicity. In contrast, conventionally etched Ti_3_C_2_T_*x*_ retains many −F terminations that can hydrolyze to release fluoride and create hydrophobic patches; such fluoride-related residues are known to induce cellular stress and toxicity. By eliminating most of −F terminations and residual etchant ions, Ti_3_C_1.5_N_0.5_T_*x*_ does not leach harmful species into the medium, and its −OH-rich surface interacts benignly with cells. Second, the Ti_3_C_1.5_N_0.5_T_*x*_ composition generates significantly less intracellular reactive oxygen species (ROS) than Ti_3_C_2_T_*x*_. Partially oxidized titanium sites (e.g., Ti­(III)) and other defects on Ti_3_C_2_T_*x*_ can catalyze ROS production, whereas Ti_3_C_1.5_N_0.5_T_*x*_ contains Ti in a higher oxidation state due to the presence of lattice nitrogen, and the surface Ti atoms are passivated by −OH and =O, so that fewer radical-generating reactions occur.

### Cell Migration, Wound Healing Potential, and In Vivo tolerance

The scratch wound assay revealed that HaCaT keratinocytes readily migrated to close the wound gap over 24 h, and this healing kinetics depended strongly on MXene dose and preparation. At 0 h, all conditions showed a clear initial gap, while by 24 h, the untreated control and low-concentration MXene groups (6.25 and 25 μg/mL) achieved nearly complete closure (SI, Figure S5). Notably, these low doses of both Ti_3_C_2_ and Ti_3_C_1.5_N_0.5_ MXenes did not visibly impede keratinocyte migration relative to control wounds, indicating minimal interference with the intrinsic wound-healing process. In contrast, the 100 μg/mL treatments resulted in significantly slower wound closure: after 24 h, scratches in high-dose MXene cultures remained only partially filled with cells, suggesting significant inhibition of cell migration and/or proliferation at this elevated concentration.

A clear dose-dependent trend emerged, with high MXene doses causing impaired wound healing compared to lower doses (Figure 12). Importantly, the extent of this impairment differed between MXene variants. Li-intercalated MXenes at 100 μg/mL showed the most pronounced reduction in wound closure, leaving wider residual gaps after 24 h. In these cultures, keratinocytes at the wound edge migrated only sparsely into the scratch area, and cell density within the wound remained low, implying that either cell motility or survival was adversely affected by the Li^+^-treated materials. By comparison, Na-intercalated MXenes at the same 100 μg/mL dose allowed significantly better gap closure. Although wound healing was still slower than in the untreated or low-dose groups, the cells exposed to Na^+^-MXene populated the wound area more extensively than those exposed to Li^+^-MXenes. This suggests that substituting Li^+^ with Na^+^ during MXene synthesis confers a notable biocompatibility advantage at high concentrations, likely by avoiding the deleterious effects of lithium on cells. Lithium ions are known to disrupt cell growth and viability at millimolar levels, so reducing residual Li^+^ in MXenes may prevent such stress on the healing monolayer.^43^

**Figure 12:**
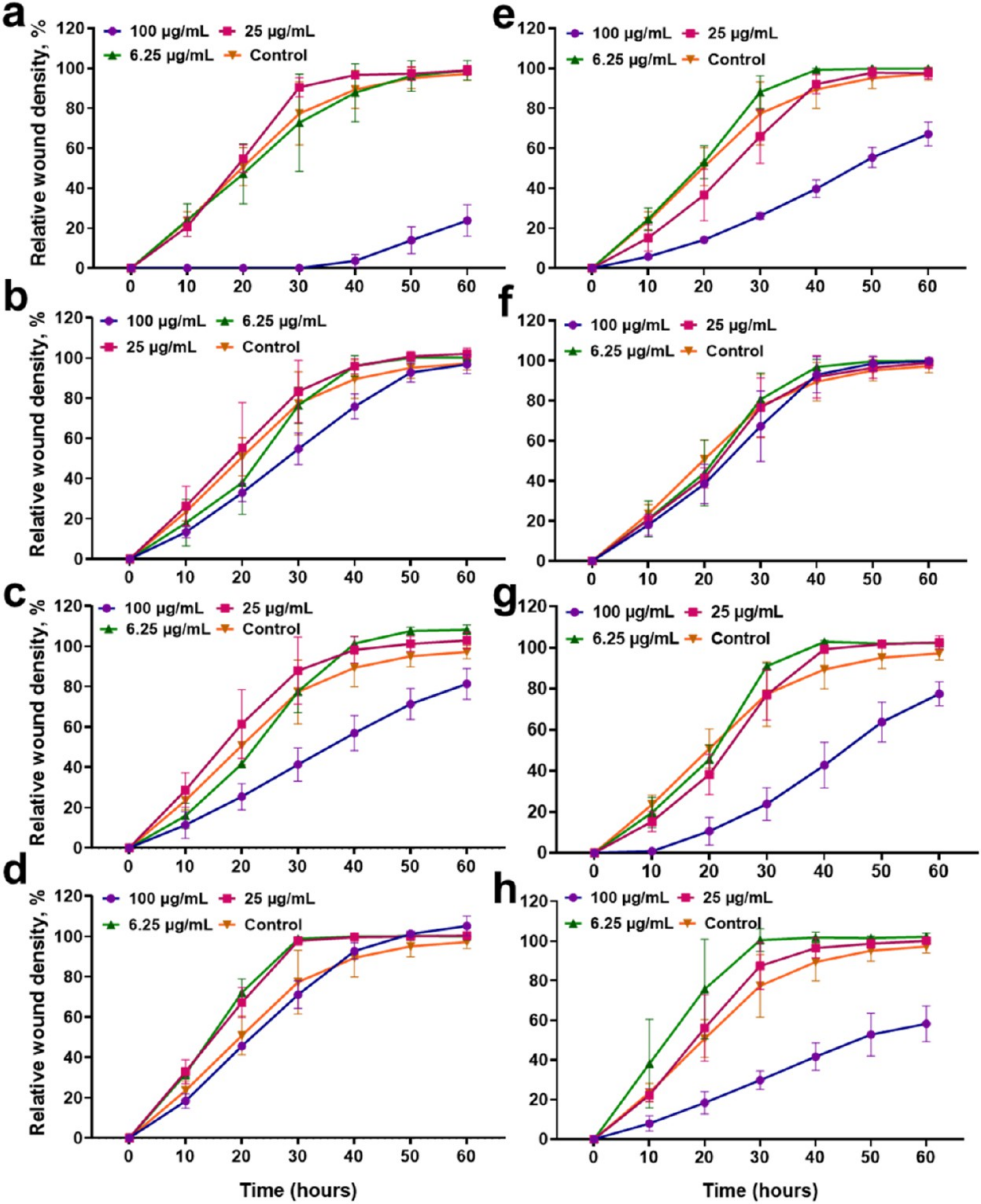
In vitro scratch wound healing assay of HaCaT keratinocytes treated with Ti_3_C_2_ and Ti_3_C_1.5_N_0.5_ MXenes, demonstrating the effects of dose, intercalant, and etching method on cell migration (*n* = 3). (a) Ti_3_C_2__Li, (b) Ti_3_C_2__Na, (c) Ti_3_C_2__H_2_O_Li, (d) Ti_3_C_2__H_2_O_Na, (e) Ti_3_C_1.5_N_0.5__Li, (f) Ti_3_C_1.5_N_0.5__Na, (g) Ti_3_C_1.5_N_0.5__H_2_O_Li and (h) Ti_3_C_1.5_N_0.5__H_2_O_Na.

Beyond intercalating cations, the MXene etching method also influenced keratinocyte wound healing. MXenes produced with an HF/HCl/H_2_O etching (water-assisted etching) step consistently supported faster scratch closure than their HF/HCl-etched counterparts. For both Ti_3_C_2_ and Ti_3_C_1.5_N_0.5_ MXenes, the inclusion of water in the etching process (which is known to modify surface terminations) led to smaller remaining wound areas at 24 h. In practical terms, HaCaT cells exposed to water-etched MXenes migrated into the wound more efficiently and formed a confluent monolayer across the scratch sooner, compared to cells exposed to MXenes prepared by the traditional HF/HCl route. This trend was evident across all tested concentrations but was most pronounced at the high dose: even though 100 μg/mL of any MXene slowed healing, the water-etched samples resulted in measurably greater wound closure than the HF/HCl-only samples at 24 h. Thus, a H_2_O-assisted synthesis improved the compatibility of MXenes with the wound-healing process, pointing to more favorable surface chemistry for cell migration.

These findings underscore the importance of MXene surface chemistry and composition in tissue regeneration contexts. The superior performance of Na-intercalated, H_2_O-etched MXenes suggests that specific surface terminations (and the absence of certain residuals) are key to preserving cell migratory capacity. Water-assisted etching likely increases the proportion of hydrophilic −OH/–O terminations on MXene surfaces while removing a significant fraction of −F terminations inherited from HF.^9,44^ Overall, the scratch assay results highlight that MXene nanomaterials can be compatible with the wound-healing process, provided they are engineered with biocompatible features. Low concentrations of Ti_3_C_2_ or Ti_3_C_1.5_N_0.5_ MXene (especially when prepared via Na^+^ and water-involved methods) do not hinder keratinocyte migration and might even allow full wound closure comparable to untreated cells. This is a promising outcome for potential applications in tissue regeneration and wound dressings, as it indicates that MXenes—known for their antimicrobial and conductive properties—could be incorporated into wound healing materials without stalling the repair of epithelial tissues.^45^ In fact, the enhanced closure seen with water-etched MXenes suggests that optimizing the synthesis can not only mitigate toxicity but possibly create surfaces that subtly promote cell migration.

### Skin Irritation Test

Histological examination of the skin at MXene injection sites revealed preserved tissue architecture across all samples in contrast to positive control—SDS injection (SI, Figure S7). The epidermis, dermis, and hypodermis remained intact and well-organized with no signs of necrosis or structural damage. At both 25 μg/mL and 100 μg/mL doses, Ti_3_C_2_T_*x*_ injections (etched via HF/HCl/H_2_O and intercalated with LiCl or NaCl) caused only minimal localized changes. Skin sections showed mild dermal edema and minimal inflammatory cell infiltrates, mainly at the higher dose, but without any substantial inflammatory areas (Figure 13a,c). Toluidine blue staining indicated no significant mast cell degranulation in these areas, consistent with a lack of an acute allergic response (Figure 13b,d). In Ti_3_C_2_T_*x*_ intercalated with NaCl treated skin, small dark aggregates of the MXene were observed within the reticular dermis (especially at 100 μg/mL); notably, these deposits were biologically inert, eliciting no surrounding tissue reaction, fibrosis, or disruption of collagen fiber organization (Figure 13c,d).

**Figure 13:**
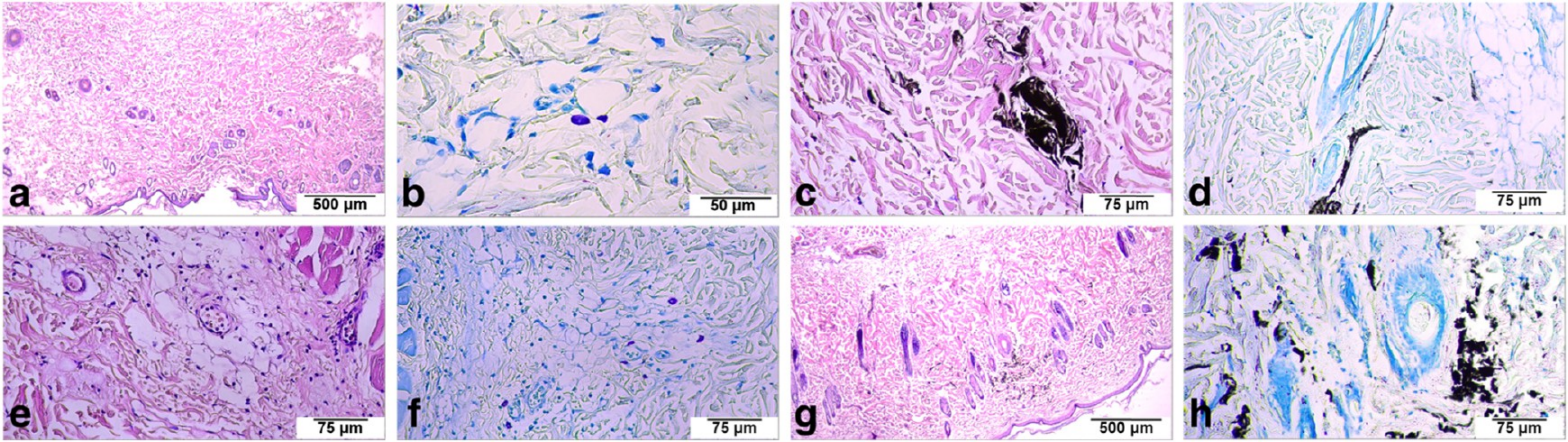
Representative histological images of skin tissues following subcutaneous injection of Ti_3_C_2_T_*x*_ and Ti_3_C_1.5_N_0.5_T_*x*_ MXenes. (a, b) Ti_3_C_2__H_2_O_Li, (c, d) Ti_3_C_2__H_2_O_Na, (e, f) Ti_3_C_1.5_N_0.5__H_2_O_Li, and (g, h) Ti_3_C_1.5_N_0.5__H_2_O_Na. The magnifications of the images are ×40 (scale bar = 500 μm; panels a and g), ×200 (scale bar = 75 μm; panels c–e), and ×300 (scale bar = 50 μm; panel b). Panels a, c, e, and g show hematoxylin and eosin (H&E) staining to assess overall tissue morphology. Panels b, d, f, and h show toluidine blue staining for mast cell evaluation.

Ti_3_C_1.5_N_0.5_T_*x*_ MXene injections elicited similarly negligible histopathological changes (Figure 13e–h). At 25 μg/mL, neither the Li^+^ nor Na^+^ intercalated Ti_3_C_1.5_N_0.5_T_*x*_ caused any detectable alteration in skin layer integrity. At the 100 μg/mL dose, the Li^+^ intercalated Ti_3_C_1.5_N_0.5_T_*x*_ sample induced a slight increase in dermal vascular caliber (vasodilation) accompanied by mild perivascular edema and a limited leukocyte infiltrate in the deep dermis/hypodermis. A modest mast cell response was noted in this group (occasional degranulated mast cells), and a transient reduction in hair follicle profiles (consistent with catagen-phase induction) was observed, indicating only a mild localized reaction. In contrast, the Na^+^-intercalated Ti_3_C_1.5_N_0.5_T_*x*_ at 100 μg/mL produced no appreciable inflammatory or structural changes apart from the presence of a few inert MXene aggregates in the dermis (similar to the Ti_3_C_2_T_*x*_ MXene Na^+^ intercalated case). Importantly, no overt chronic inflammation or tissue damage was evident in any MXene-treated skin, underscoring that all four MXene formulations were well-tolerated in vivo at the tested doses.

It is plausible that MXenes with other C/N ratios could yield different or potentially enhanced biological performance due to variations in electronic structure or surface terminations. Carbonitrides can also be produced with Ti_2_(C,N) and Ti_4_(C,N)_3_ structures,^18^ but biocompatibility and biomedical applications of those compositions remain unexplored. Future work could certainly explore the effect of varying C/N ratios in more detail, provided the appropriate MAX phase precursors and synthesis routes become available. We hope that our work demonstrating the excellent biocompatibility of this particular carbonitride will inspire systematic studies of other MXenes in the Ti–N–C system.

## Conclusions

This study demonstrates that the biocompatibility of Ti_3_C_2_T_*x*_ and Ti_3_C_1.5_N_0.5_T_*x*_ MXenes can be precisely modulated by tailoring synthesis parameters, particularly etching conditions and intercalant choice. Surface termination profiles—shaped by etchant chemistry and delamination route—strongly influence cytotoxicity, oxidative stress, inflammatory signaling, wound healing, and skin tolerance in vivo. Specifically, Ti_3_C_1.5_N_0.5_T_*x*_ exhibited consistently higher biocompatibility than Ti_3_C_2_T_*x*_, while more dilute etchants combined with Na^+^ intercalation yielded MXenes with reduced −F terminations, enhanced hydroxylation, and improved biological performance. At subcytotoxic doses (≤25 μg/mL), these formulations preserved keratinocyte viability, supported wound closure, and triggered negligible oxidative or inflammatory responses in vitro. Importantly, in vivo histological analysis confirmed the absence of acute skin toxicity across all tested MXene types and concentrations. No structural damage, inflammatory infiltration, or mast cell activation was observed in the dermis or hypodermis, even in the presence of dermal MXene aggregates. These findings not only underscore the importance of surface terminations (−F vs −OH) and residual intercalants in dictating MXene bioresponse but also highlight the critical role of particle size and colloidal stability in modulating cell–MXene interactions. By tuning these parameters, it is possible to design MXenes that retain their functional properties while minimizing cytotoxicity and inflammation. Such optimized MXenes hold strong promise for safe integration into biomedical applications, as evidenced by the negligible cytotoxicity and lack of acute inflammatory response observed for our most refined formulations.

## Experimental Section

### MXene Synthesis

MAX phases, Ti_3_AlC_2_ (Carbon Ukraine), and Ti_3_AlC_1.5_N_0.5_ (made at Drexel University) were washed before etching to eliminate intermetallic impurities. This was done by intensive powder mixing with 9 M HCl (1 g MAX phase: 10 mL HCl) at room temperature under an ice bath for 18 h, followed by powder washing to almost neutral pH and drying. Ti_3_C_2_ and Ti_3_C_1.5_N_0.5_ were conventionally synthesized by a wet chemical etching method. Acids (49 wt %), HF (Acros Organics), 12 M HCl (Fisher Scientific), and deionized (DI) water with electrical resistivity 15 MΩ·cm were used. The dried MAX phase precursors were mixed with HF/HCl (2:18) and HF/HCl/H_2_O (2:12:6) solutions in plastic bottles of 250 mL, respectively, for 24 h at 35 °C and 300 rpm of stir bar rotation. The etched content was washed multiple times with distilled water using a centrifuge, 3500 rpm for 10 min, until the pH reached ∼ 6. More washing cycles were performed with the HF/HCl batches due to a more acidic environment than the HF/HCl/H_2_O batches. The etched and washed multilayered MXenes were split in half and transferred to clean plastic bottles. Multilayered MXenes were intercalated based on 1 g of LiCl or NaCl per 1 g of Ti_3_AlC_2_ MAX, dissolved in 50 mL of DI water, and stirred at 100 rpm at room temperature for 24 h. The washing procedure was again performed to increase pH from ∼ 2.5 to ∼ 6.5, using centrifugation at 3500 rpm for 15 min. Some supernatants were dark after regular centrifugation, some needed 5 min shaking in a shaker, and some required an additional 10 min sonication. The summary of all eight Ti_3_C_2_ and Ti_3_C_1.5_N_0.5_ supernatants obtained after NaCl and LiCl intercalation is given in SI Table S2. Each batch of collected supernatant was centrifuged at 3500 rpm for 30 min to obtain supernatant for the final use.

### DLS, UV–vis–NIR, and FTIR Spectroscopy

The flake size and distribution, followed by zeta potential, were measured by dynamic light scattering (MALVERN Instruments Zetasizer) at concentrations of about 0.01 mg/mL. The optical properties of Ti_3_C_2_ and Ti_3_C_1.5_N_0.5_ were evaluated by UV–vis–NIR spectroscopy (Thermo Scientific Evolution 201 spectrometer) and Fourier transform infrared spectroscopy, FTIR (Invenio-X, Bruker, Germany). Very diluted suspensions obtained from the final supernatants were used for both measurement techniques. Supernatant concentrations were calculated from the plasmonic absorption of UV–vis–NIR spectra collected in transmission mode from 200 to 1000 nm. The FTIR measurements were performed in total reflectance mode, whereas the measurements were taken at room temperature and average humidity. The gold-coated mirror was used as a reference background, and the background was respectively subtracted for each sample in the 1 to 25 μm range.

### Scanning Electron Microscopy

The morphology, structural features, and elemental composition of the synthesized MXenes were analyzed using a scanning electron microscope (Jeol 7001TTLS) integrated with an energy-dispersive X-ray spectroscopy system.

### Atomic Force Microscopy

AFM measurements were carried out using an ICON (Bruker) system, and the profilometric (Z-sensor) data were analyzed with the Gwyddion software.

### Raman spectroscopy

Raman spectroscopy was conducted using a Renishaw system equipped with a microscope enclosure, a 633 nm laser source, and a ×50 Leica objective lens. Spectra were acquired with five accumulations, each with an exposure time of 0.1 s, using 0.5% of the total laser power.

### X-ray Photoelectron Spectroscopy

Samples for X-ray photoelectron spectroscopy (XPS) experiments were prepared by dropping MXene solution on a silicon wafer. The solvent was evaporated at room temperature, resulting in the formation of a homogeneous layer of MXene on the Si surface. The XPS measurements were done using the AXIS Supra+ instrument (Kratos Analytical) equipped with a monochromatic Al K_α_ X-ray source (*h*ν = 1486.6 eV, operating at 10 mA, 15 kV). The system base pressure was equal to *p*_b_ = 3.1 × 10^–9^ Torr. The pass energy was equal to 160 eV (scanning step 0.9 eV) or 20 eV (scanning step 0.05 eV) for survey spectra and high-resolution spectra, respectively. For the compensation of the charging effect, the Kratos charge neutralizer system was used. The binding energy scale was calibrated with respect to the C–C component of C 1s spectra (284.8 eV). The acquired spectra were analyzed using CASA XPS software and embedded algorithms. The components of the high-resolution spectra were presented with Gaussian (70%) and Lorentzian (30%) lines, while the background was with Shirley’s function.

### Reactive Oxygen Species

The ROS formation was measured in human keratinocyte cells (HaCaT, passage 10, CLS, Eppelheim, Germany) using a dye, 2′,7′-dichlorodihydrofluorescein diacetate (H2DCFDA). The cells with a density 3 × 10^4^ per cm^2^ were plated in a black 96-well plate and cultivated in Dulbecco’s modified Eagle’s medium (CLS) supplemented with 10% fetal bovine (FBS; Sigma-Aldrich), 100 U/mL penicillin, and 100 μg/mL streptomycin (Gibco, Grand Island, NY) under standard conditions overnight. MXenes in different concentrations (100, 25, and 6.25 μg/mL) were added to the plate for 2, 4, 6, and 24 h incubation. After the incubation period, cells were washed with PBS and then incubated with 10 μM H2DCFDA in the respective medium. Positive control cells were stimulated with 3 mM H_2_O_2_. The fluorescence signal was recorded using an Infinite 200 PRO (Tecan, Männedorf, Switzerland) microplate reader at an excitation wavelength of 485 nm and an emission wavelength of 528 nm. For each sample, there were 3 repetitions.

### Cytotoxicity and Live/Dead Staining

The immortalized human keratinocyte cells (HaCaT, passage 10) were obtained from the Cell Lines Service GmbH (catalog no.300493, Eppelheim, Germany) and were used to assess the cytotoxicity of titanium carbide and titanium carbonitride MXenes. HaCaT cells were selected as the in vitro model due to their widespread use in dermatological and toxicological research. They closely mimic normal human epidermal keratinocytes in morphology, proliferation, and differentiation capacity, making them a suitable and reproducible model for assessing cellular responses to nanomaterials. Given the growing interest in applying MXenes to skin-related biomedical applications, including wound healing, tissue regeneration,^45^ and photothermal therapy of skin malignancies,^46^ the HaCaT model offers physiologically relevant insights. Moreover, HaCaT cells are considered a reliable proxy for general epithelial toxicity screening, providing an initial biocompatibility assessment that may be extrapolated to other epithelial or mucosal systems. Cells were cultivated in Dulbecco’s modified Eagle’s medium (CLS) supplemented with 10% fetal bovine serum (FBS; Sigma-Aldrich), 100 U/mL penicillin, and 100 μg/mL streptomycin (Gibco, Grand Island, NY). Cell lines were authenticated by STR profiling (CLS), confirmed to have the correct morphology, and were negative for mycoplasma. For the experiment, cells were maintained in 75 cm^2^ culture flasks under standard conditions (humidified air with 5% CO_2_ at 37 °C), with the medium refreshed every 2–3 days. Subsequently, HaCaT cells were seeded in a 96-well plate (Sarstedt) at a density of 15,000 cells/cm^2^, using 200 μL of complete medium per well. After overnight incubation under standard conditions, MXenes at different concentrations (100, 50, 25, 12.5, and 6.25 μg/mL) were added to the culture plate. The cells were incubated with the materials for 24 h. After incubation, the cells were gently washed twice with phosphate-buffered saline (PBS) to ensure the complete removal of any residual materials. After a 72-h incubation period, the metabolic activity was evaluated by adding a resazurin solution (0.15 mg/mL, pH 7.4) at a volume equivalent to 10% of the culture medium in each well. The Resazurin Reduction assay assessed cell proliferation on the third and sixth days. Positive controls (cells cultured without materials) and negative controls (medium only) were included in the resazurin reduction assay analysis. The plate with resazurin solution was incubated for 2 h under standard culture conditions (humidified air with 5% CO_2_ at 37 °C). The fluorescence intensity was measured using an Infinite 200 PRO (Tecan, Männedorf, Switzerland) microplate reader with the optimal λ_Ex_ = 530 nm and λ_Em_ = 590 nm for resorufin. For each sample, there were 3 repetitions.

Likewise, cell viability was assessed using a fluorescent live/dead staining after the sixth day of incubation. The viability assay was performed using Calcein AM (ThermoFisher Scientific) for live cells (green fluorescence) and Propidium Iodide (Sigma-Aldrich) for dead cells (red fluorescence). The staining solution was prepared according to the manufacturer’s protocol and incubated with the cells for minutes at 37 °C in the dark.

Fluorescence imaging was performed using a Leica Fluorescence Microscope DMI4000B (Leica Microsystems, Germany) at 10× magnification (scale bar = 100 μm). Images were acquired in the FITC and TRITC channels for green (live) and red (dead) fluorescence signals, respectively.

### Apoptosis/Necrosis

The cytotoxicity of the MXene samples was evaluated using the Annexin V and propidium iodide (PI) apoptosis assay after 24 h of treatment. HaCaT cells were seeded in a 25 cm^2^ flask at a density of 1 × 10^5^ cells/cm^2^ and cultured in DMEM supplemented with 10% FBS, 4.5 g/L glucose, and 4 mM l-glutamine for 24 h at 37 °C in a humidified atmosphere containing 5% CO_2_. The cells were then treated with all types of MXene samples at a concentration of 25 μg/mL for 24 h. Untreated cells served as the negative control.

After incubation, the cells were washed with PBS, harvested, and resuspended in a binding buffer. The difference with the untreated cells was counted as dead cells. They were then incubated with Annexin V (1:20 v/v; ImmunoTools, Germany) for 20 min at 4 °C. Subsequently, the cells were washed with a binding buffer and stained with PI (1:40 v/v; CAS 25535–16–4, Sigma-Aldrich). The stained samples were analyzed using a FlowSight Imaging Flow Cytometer (Amnis, part of Merck Millipore), and data processing was performed with IDEAS Software version 6 (Amnis, part of Merck Millipore).

### ELISA

To test the immunomodulatory properties of MXenes, culture media were collected from immortalized human keratinocyte (HaCaT) cell cultures after 24 h of incubation under standard conditions (humidified air with 5% CO_2_ at 37 °C) with MXenes solution at a concentration of 25 μg/mL. Before the sample addition step in ELISA immunoassays, the samples were diluted 1:4 in 1% BSA solution.

The immune response and inflammatory reactions of the cells were determined using an ELISA assay. Reagents and protocols from R&D Systems were used. A 96-well plate was prepared with 100 μL of IL-6 and IL-8 Capture antibodies per well. The plate was sealed with a cover film and incubated at room temperature overnight. After incubation, the solution was discarded, and wells were washed three times with 400 μL of ELISA wash buffer (0.05% Tween-20 in PBS). The plates were blocked by adding 300 μL of 1% BSA and incubated at room temperature for 1 h, followed by washing with ELISA wash buffer. Then, 100 μL of each diluted sample was added to the wells, using duplicates for IL-6 and IL-8 standards. The plates were sealed and incubated at room temperature for 2 h, followed by washing with ELISA wash buffer. Next, 100 μL of the appropriate detection antibody, diluted in 1% BSA (167 μL DetAB + 9.833 mL 1% BSA), was added to each well. The plates were sealed and incubated at room temperature for 2 h, followed by washing with ELISA wash buffer. Next, 100 μL of Streptavidin-HRP solution (diluted 1:40 in 1% BSA) was added to each well and incubated for 20 min at room temperature, protected from light, followed by washing with ELISA wash buffer.

Substrate solutions A and B were mixed at a 1:1 ratio immediately before use, and 200 μL of the mixture was added to each well for 30 min, protected from light. After incubation, 50 μL of stop solution was added, ensuring thorough mixing of the solution. The optical density of the samples was measured at a wavelength of 450 nm using an Infinite 200 PRO (Tecan, Männedorf, Switzerland) microplate reader, with a wavelength correction set to 540 or 570 nm.

### Scratch Test

To evaluate the effect of MXenes on HaCaT viability, a scratch test was performed to detect the migration ability and invasion ability of human keratinocyte cells. For this experiment, the cells were incubated for 24 h with MXenes at different concentrations (100, 25, and 6.25 μg/mL). When cell density reached about 100 % confluence in a 96-well Incucyte Imagelock Plate, the single layer was scraped using the Woundmaker (Sartorius) to simultaneously create a cell-free zone in all wells. After wounding, the medium was aspirated from each well, and the cells were thoroughly washed twice with Dulbecco’s phosphate-buffered saline (DPBS). After washing, 100 μL of culture media was added to each well, and the cell plate was placed into the Incucyte Live-Cell Analysis System and the plate was allowed to warm to 37 °C for 30 min before scanning. Scans were performed every 2 h for 60 h.

### Skin Irritation Test

The animal study was approved by the Ethics Committee of Sumy State University. The study adhered to the guidelines outlined in the Handbook for the Care and Use of Laboratory Animals (1996) and Directive 2010/63/EU of the European Parliament and Council on protecting animals used for scientific purposes (2010).

A total of 5 male white rats C57BL/6 line bought from Biomodelservice, with an average weight of 250–350 g, were selected for the skin irritation study. All animals were housed in controlled conditions—plastic cages with pine-sawdust flooring, maintained at 24 °C, with a 12 h light-dark cycle and ad libitum access to water and Biobased commercial food.

Each rat served as a representative of each type of MXene in two concentrations: 100 and 25 μg/mL. Rats that were administered with 2% sodium dodecyl sulfate (SDS) and 0.9% NaCl solutions served as positive and negative controls (SI, Figure S6). Each solution was prepared under sterile conditions and diluted in distilled water in 1.5 mL Eppendorf tubes. Prior to the dilution operation, MXenes were sonicated at 35W, 50 Hz for 20 min to disperse the fractions uniformly.

To monitor the tissue’s reaction to the MXenes, an intradermal injection was administered to the dorsal area using a modified skin irritation protocol as described in.^47^ The area on the back of the rats was shaved and sterilized with 70% ethanol. The test solutions were applied the next day to minimize the effect of shaving irritation. Using insulin syringes U-100 30G 1 mL/cc 5/16", 50 μL of solutions were intracutaneously injected into various back skin regions that were spaced apart by roughly 1–2 cm. Skin observations were made at 30 min, 1 h, 24 h, and 7 days following the injection to track the rate of inflammation. The animals were sedated with 80 μL of “Prosedan” drug, which was injected intramuscularly into the hip, and 2% lidocaine was applied to the papules’ periphery before each processional. Skin cuts were used to collect the skin sample punched for the biopsy. After preserving the tissue by immersing the samples in 4% formaldehyde, the samples were transported to the CSD laboratory (Kyiv, Ukraine) for histological analysis.

### Histology

Skin samples were collected from the injection sites to assess structural changes in the cutaneous tissues. Immediately after resection, the samples were fixed in 10% neutral buffered formalin for 24 h. Following fixation, they were processed using standard protocols with an automated tissue processor (Milestone LOGOS Microwave Hybrid Tissue Processor, Milestone, Italy). During paraffin embedding, the samples were oriented to allow evaluation of all skin layers. Paraffin-embedded blocks were sectioned at a thickness of 4 μm using a Thermo Scientific HM 340E microtome. Sections were stained with hematoxylin and eosin (H&E) using the Dako CoverStainer (Agilent) for routine histological examination. Additionally, toluidine blue staining was performed to assess mast cell activity. Histological analysis was conducted via light microscopy to evaluate tissue alterations, vascular responses, mast cell distribution and degranulation, as well as signs of inflammation or tissue repair.

### Statistics

The results were analyzed statistically using the GraphPad Prism 9.1.1 software package. All experiments were executed in triplicate, and the outcomes are presented as mean ± standard deviation. Significance levels were determined using a one-way analysis of variance (*p* < 0.05 denoting significance)

## Supplementary Material



## Data Availability

The raw/processed data required to reproduce these findings cannot be shared at this time, as the data also forms part of an ongoing study. The raw data are available on request.

## Notes

AFM images of Ti_3_C_2_ and Ti_3_C_1.5_N_0.5_ samples (Figure S1); SEM images of Ti_3_C_2_ and Ti_3_C_1.5_N_0.5_ samples (Figure S2); EDX spectra of Ti_3_C_2_ and Ti_3_C_1.5_N_0.5_ samples (Figure S3); XPS high-resolution spectra of F 1s (a) and Cl 2p (b) energy regions (Figure S4); in vitro scratch wound healing assay of HaCaT keratinocytes (Figure S5); visual representation of papules formed after administration of test solutions (Figure S6); skin reaction to NaCl and SDS2 injections (Figure S7); physical observations of sediments and Ti_3_C_2_ and Ti_3_C_1.5_N_0.5_ supernatants (Table S1); overview of flake size and zeta potential values (Table S2); Summary of MXene biocompatibility and ROS production (Table S3) (PDF)
