# Supplementary material for: Effects of Etching and Delamination on Biocompatibility of Ti-Based MXenes
Source: ACS Appl Mater Interfaces. 2025 Aug 18;17(34):47919–37. doi: 10.1021/acsami.5c08807 (PMC12400265; doi:10.1021/acsami.5c08807)
Supplement: Supplementary file 1 [file am5c08807_si_001.pdf]

SUPPORTING INFORMATION

Effects of Etching and Delamination on Biocompatibility of Ti-Based MXenes

Kateryna Diedkova[1,2*], Iryna Roslyk[3], Nikola Kanas[4], Lita Grine[2], Volodymyr Deineka[1,2], Agata Blacha-Grzechnik[5,6], Martins Boroduskis[2], Igor Iatsunskyi[7], Błażej Anastaziak[7], Anastasia Konieva[1,8], Pavlo Shubin[1,2], Wojciech Simka[5], Marks Truhins[2], Oksana Sulaieva[9], Ilya Yanko[1], Veronika Zahorodna[10], Goran Stojanovic[4], Oleksiy Gogotsi[1,10], Yury Gogotsi[1,3*], Maksym Pogorielov[1,2*]

[1]Biomedical Research Centre, Sumy State University, 40007 Sumy, Ukraine
[2]University of Latvia, LV-1004 Riga, Latvia
[3]A. J. Drexel Nanomaterials Institute, and Department of Materials Science and Engineering, Drexel University, Philadelphia, Pennsylvania 19104, USA
[4]University of Novi Sad, BioSense Institute, Zorana Dindica 1, 21000 Novi Sad, Serbia
[5]Faculty of Chemistry, Silesian University of Technology, Strzody 9, 44-100, Gliwice, Poland
[6]Centre for Organic and Nanohybrid Electronics, Silesian University of Technology, Konarskiego 22B, 44-100, Gliwice, Poland
[7]NanoBioMedical Centre, Adam Mickiewicz University, 3, Wszechnicy Piastowskiej Str., 61-614 Poznan, Poland
[8]Department of Anatomy, University Hospital Essen, Hufelandstraße 55, Essen, 45147, Germany
[9]Medical Laboratory CSD, 45 Vasylkivska St, Kyiv 02000, Ukraine
[10]Materials Research Centre, 3 Krzhizhanovskogo Str., 03142 Kyiv, Ukraine.

*Corresponding authors: kateryna.diedkova@lu.lv, gogotsi@drexel.edu, maksym.pogorielov@lu.lv

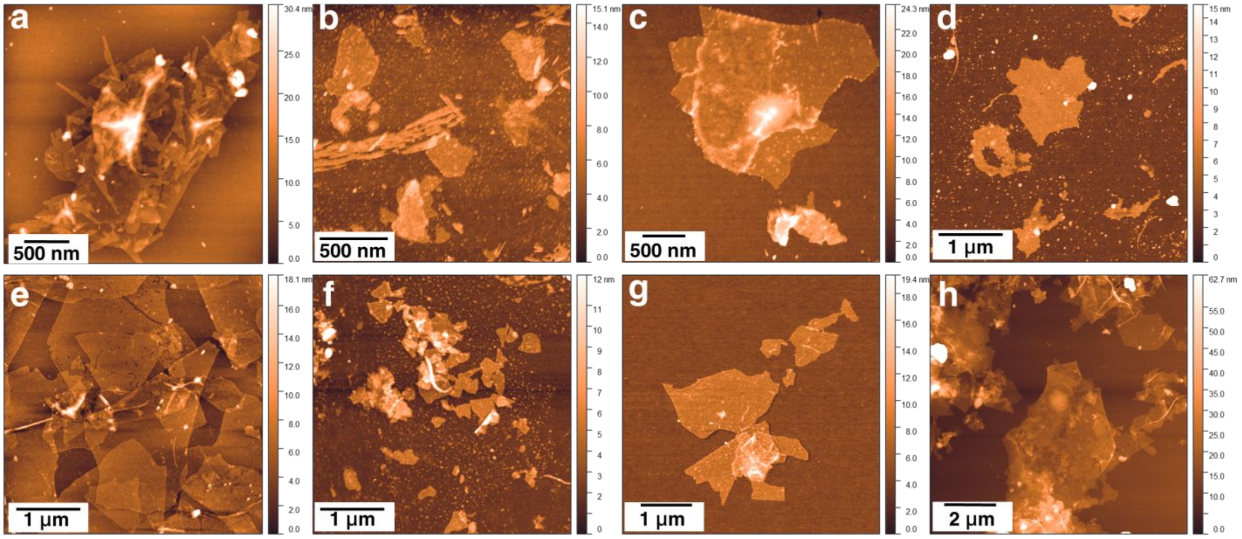

Supplement Figure S1. AFM images of Ti_3C_2 and $Ti_3C_{1.5}N_{0.5}$ samples, where (a) Ti_3C_2_Li, (b) Ti_3C_2_Na, (c) Ti_3C_2_H_2O_Li, (d) Ti_3C_2_H_2O_Na, (e) $Ti_3C_{1.5}N_{0.5}$_Li, (f) $Ti_3C_{1.5}N_{0.5}$_Na, (g) $Ti_3C_{1.5}N_{0.5}$_H_2O_Li, and (h) $Ti_3C_{1.5}N_{0.5}$_H_2O_Na.

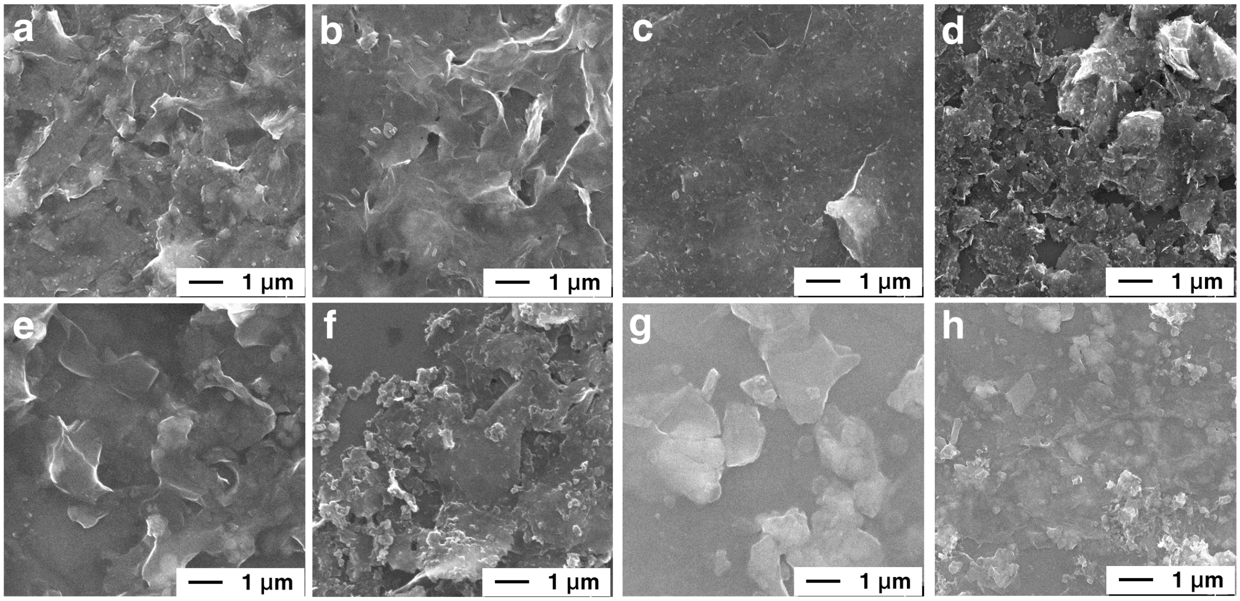

Supplement Figure S2. SEM images of Ti_3C_2 and $Ti_3C_{1.5}N_{0.5}$ samples, where (a) Ti_3C_2_Li, (b) Ti_3C_2_Na, (c) Ti_3C_2_H_2O_Li, (d) Ti_3C_2_H_2O_Na, (e) $Ti_3C_{1.5}N_{0.5}$_Li, (f) $Ti_3C_{1.5}N_{0.5}$_Na, (g) $Ti_3C_{1.5}N_{0.5}$_H_2O_Li, and (h) $Ti_3C_{1.5}N_{0.5}$_H_2O_Na.

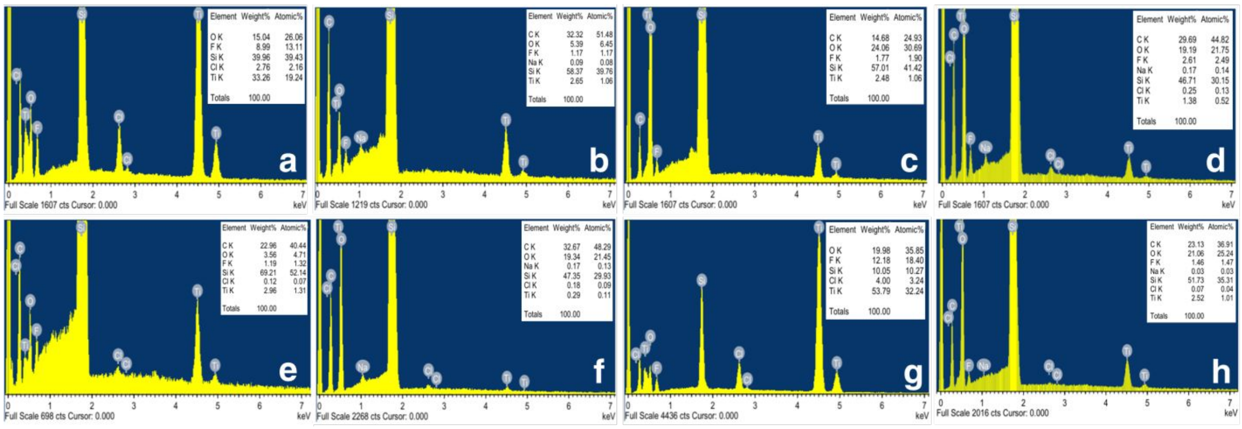

Supplement Figure S3. EDX spectra of Ti_3C_2 and $Ti_3C_{1.5}N_{0.5}$ samples, where (a) Ti_3C_2_Li, (b) Ti_3C_2_Na, (c) Ti_3C_2_H_2O_Li, (d) Ti_3C_2_H_2O_Na, (e) $Ti_3C_{1.5}N_{0.5}$_Li, (f) $Ti_3C_{1.5}N_{0.5}$_Na, (g) $Ti_3C_{1.5}N_{0.5}$_H_2O_Li, and (h) $Ti_3C_{1.5}N_{0.5}$_H_2O_Na.

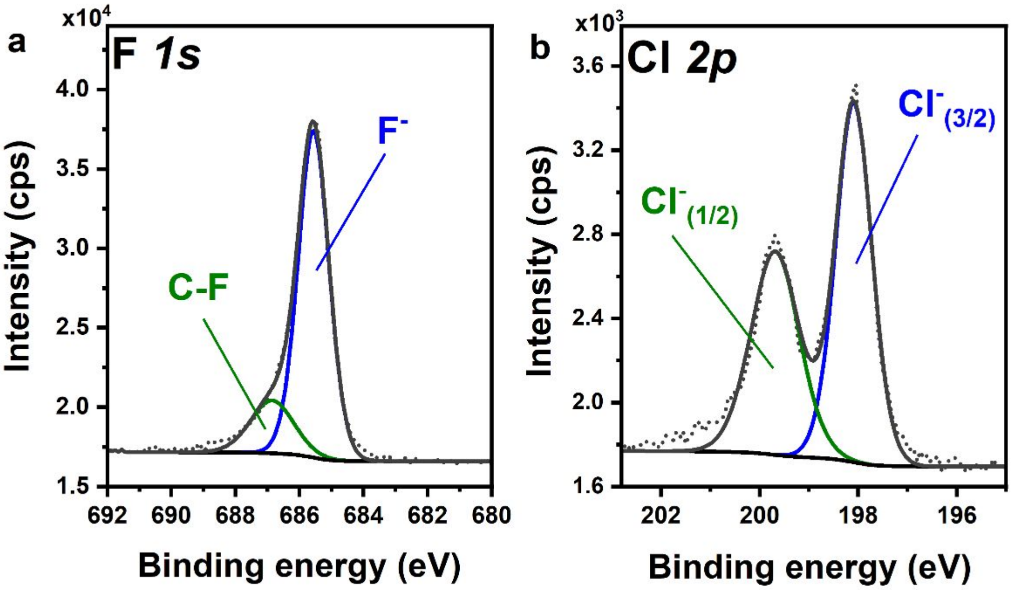

Supplement Figure S4. XPS high-resolution spectra of F 1s (a) and Cl 2p (b) energy regions recorded for Ti_3C_2_H_2O_Li.

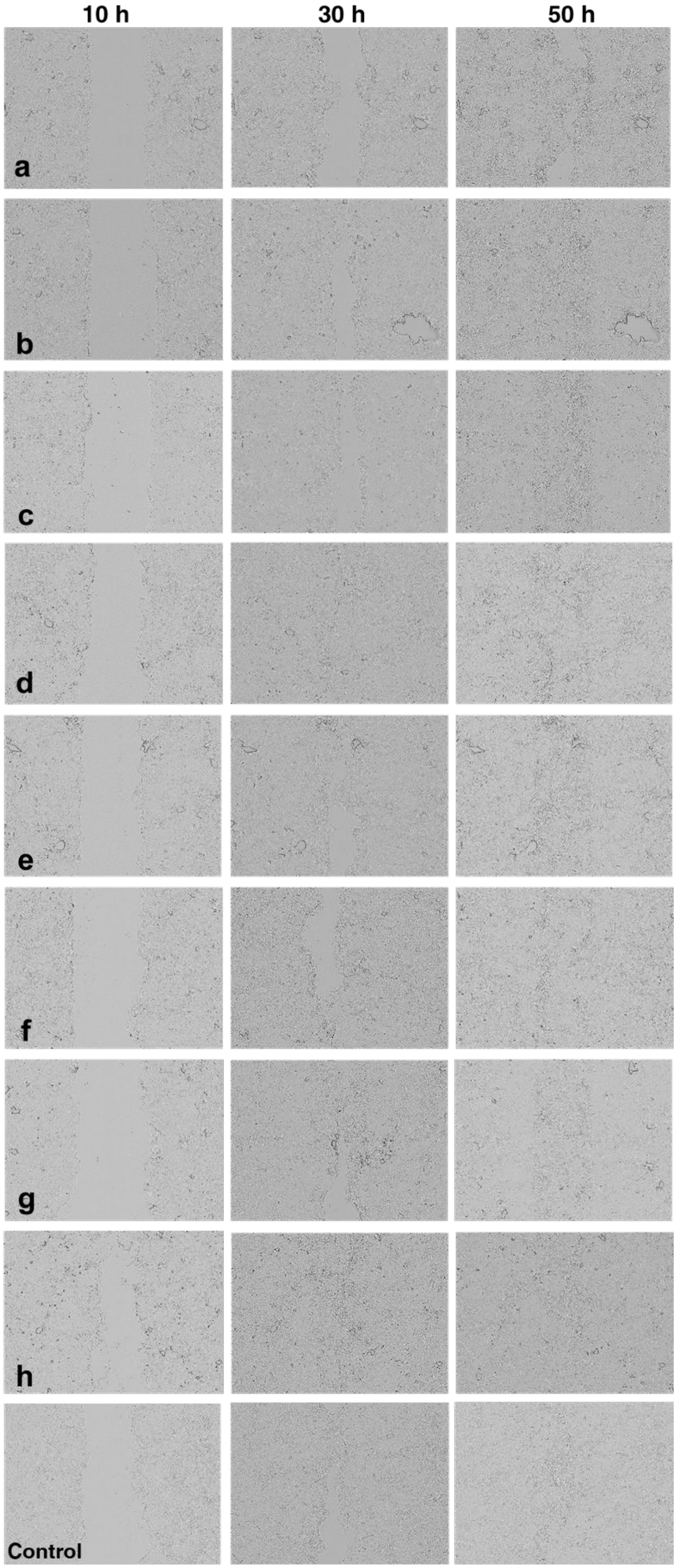

Supplement Figure S5. In vitro scratch wound healing assay of HaCaT keratinocytes treated with Ti_3C_2 and $Ti_3C_{1.5}N_{0.5}$ MXenes, demonstrating the effects of dose, intercalant, and etching method on cell migration. (a) Ti_3C_2_Li, (b) Ti_3C_2_Na, (c) Ti_3C_2_H_2O_Li, (d) Ti_3C_2_H_2O_Na, (e) $Ti_3C_{1.5}N_{0.5}$_Li, (f) $Ti_3C_{1.5}N_{0.5}$_Na, (g) $Ti_3C_{1.5}N_{0.5}$_H_2O_Li and (h) $Ti_3C_{1.5}N_{0.5}$_H_2O_Na.

Type of MXene	Concentrations	0 min	5 min	60 min
Ti_3C_2_H_2O_Li	100 and 25 µg/mL	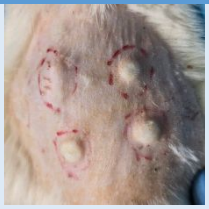	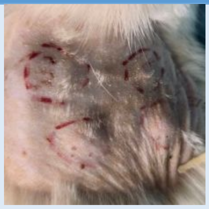	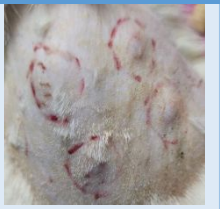
Ti_3C_2_H_2O_Na	100 and 25 µg/mL	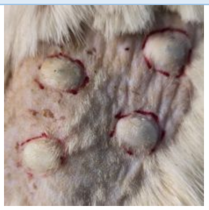	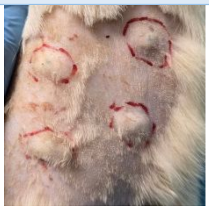	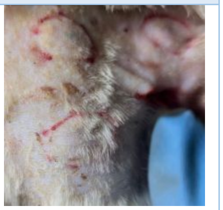
$Ti_3C_{1.5}N_{0.5}$_H_2O_Li	100 and 25 µg/mL	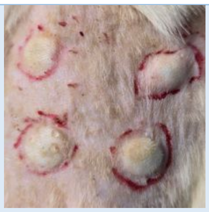	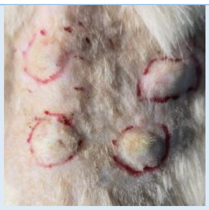	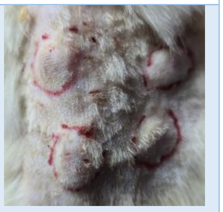
$Ti_3C_{1.5}N_{0.5}$_H_2O_Na	100 and 25 µg/mL	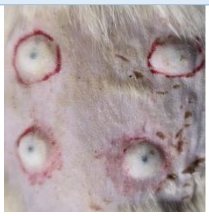	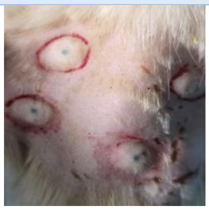	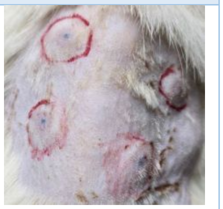
SDS	2%	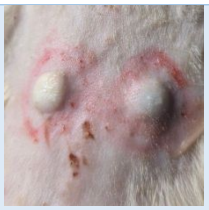	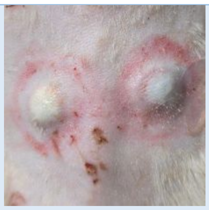	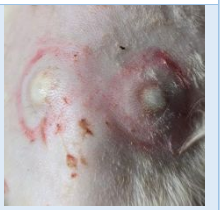
NaCl	0.5%	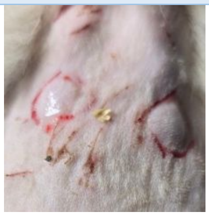	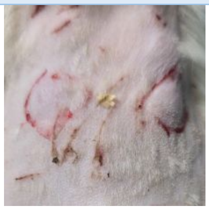	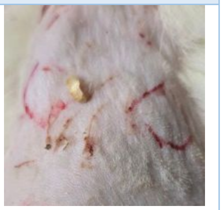

Supplement Figure S6. Visual representation of papules formed after administration of test solutions after 0 min, 5 min and 60 min.

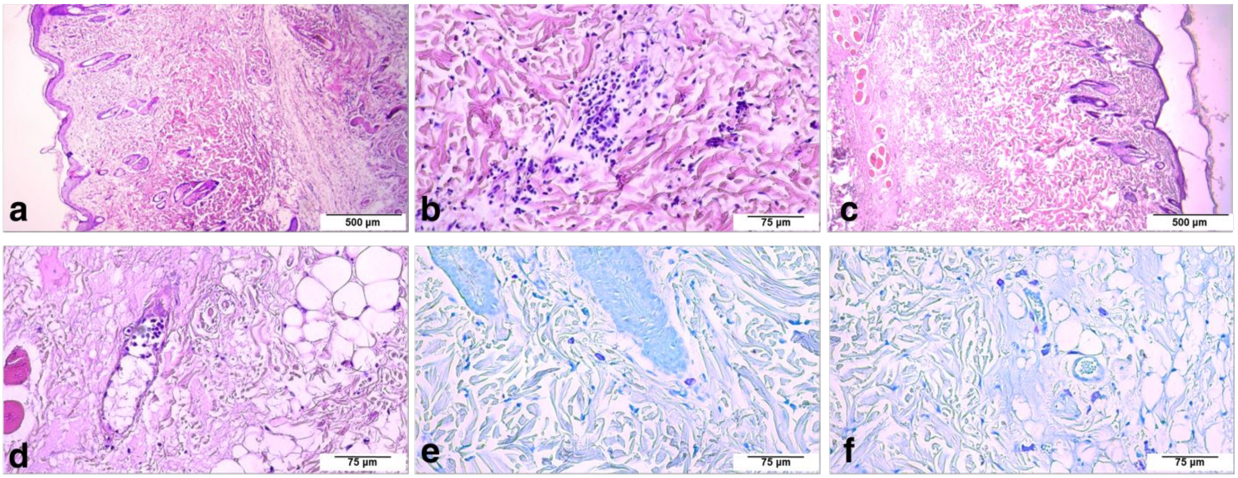

Supplement Figure S7. Skin reaction to NaCl and SDS2 injections. (a) and (b) show skin structure after NaCl application. After NaCl injection, the skin demonstrated preserved histoarchitecture and normal structure of epidermis, dermis and hypodermis with a mild edema and local infiltrates in the deep layers of dermis and hypodermis; (c-f) skin structure after injection of SOS 2%. This intervention was associated with prominent vasodilatation and edema in dermis and hypodermis; (c), collagen fibers swelling and diffuse inflammatory infiltration (d). These changes were accompanied by catagen induction in hair follicles (e) and reaction of mast cells with degranulation features (f).

Supplement Table 1. Physical observations of sediments and Ti_3C_2 and $Ti_3C_{1.5}N_{0.5}$ supernatants at each processing stage.

Material	Etchant	Intercalant	Sediment	Supernatant after		
				centrifugation	shaking	sonication
Ti_3C_2	HF/HCl (2:18)	NaCl	swelled	dark	dark & thick	-
		LiCl	swelled	dark	dark & thick	-
	$HF/HCl/H_2O$ (2:12:6)	NaCl	semi-swelled	transparent	semi-transparent	dark
		LiCl	swelled	dark	dark & thick	-
$Ti_3C_{1.5}N_{0.5}$	HF/HCl (2:18)	NaCl	swelled	semi-transparent	dark & thick	-
		LiCl	swelled	dark	dark & thick	-
	$HF/HCl/H_2O$ (2:12:6)	NaCl	not swelled	transparent	semi-transparent	dark
		LiCl	swelled	dark	dark & thick	-

Supplement Table 2. Overview of flake size and zeta potential values.

MXene	Etchant	Size (nm)		Zeta potential (mV)	
		**LiCl**	**NaCl**	**LiCl**	**NaCl**
**Ti_3C_2**	**HCl/HF**	1380	1607	- 51.4	- 49.7
	**$HF/HCl/H_2O$**	1600	266	- 51.6	- 55.7
**$Ti_3C_{1.5}N_{0.5}$**	**HCl/HF**	1837	1696	-48.4	-50.2
	**$HF/HCl/H_2O$**	1627	283	-52.2	-51.9

Supplement Table 3. Summary of MXene biocompatibility and ROS production.

MXene	Etching	Inter-calation	Biocompa-tible dose, 3 days (µg/mL)	Biocompa-tible dose, 6 days (µg/mL)	Flow cytometry (% living cells)	Maximum % of ROS (DCF) after 25 µg/mL
**Ti_3C_2**	**HF/HCl**	**LiCl**	100	6.25	24.2	135.30
		**NaCl**	100	50	83.54	136.62
	**$HF/HCl/H_2O$**	**LiCl**	12.5	12.5	36.4	129.58
		**NaCl**	12.5	12.5	62.2	115.29
**$Ti_3C_{1.5}N_{0.5}$**	**HF/HCl**	**LiCl**	100	12.5	53.7	128
		**NaCl**	100	25	87.9	112.89
	**$HF/HCl/H_2O$**	**LiCl**	12.5	12.5	60.3	141.60
		**NaCl**	25	25	56.1	84.01
